# Assessing the diagnostic accuracy of postnatal clinical scoring methods and foot length measurement for estimating gestational age and birthweight of newborns in low- and middle-income countries: a systematic review and meta-analysis

**DOI:** 10.1136/bmjpo-2024-002717

**Published:** 2024-08-30

**Authors:** Shiyam Sunder Tikmani, Thomas Mårtensson, Sumaira Khalid, Muhammad Uzair, Qammerulanissa Ali, Anum Rahim, Andreas Mårtensson, Sarah Saleem, Nick Brown

**Affiliations:** 1 Global health and migration unit, Department of Women’s & Children’s Health, Uppsala University, Uppsala, Sweden; 2 Population and Reproductive Health Section, Department of Community Health Sciences, Aga Khan University, Karachi, Pakistan; 3 Department of Public Health, College of Health Professions Marshall University, Huntington, West Virginia, USA; 4 Epidemiology and Biostatistic Section, Department of Community Health Sciences, Aga Khan University, Karachi, Pakistan

**Keywords:** Neonatology

## Abstract

**Background:**

This study aimed to update systematic reviews and meta-analyses on the diagnostic accuracy of postnatal clinical scoring (PCS) methods and foot length (FL) measurement for assessing gestational age (GA) and birth weight in low-income and middle-income countries (LMICs). In addition, the quality of reference standards, including antenatal ultrasound (A-US), last menstrual period (LMP), PCS and newborn weighing scales, was also evaluated.

**Methods:**

Studies from LMICs published between January 2000 and February 2024 were searched, using databases such as PubMed, Web of Science, Cochrane Library, CINAHL and Scopus. Studies that compared PCS and/or FL with LMP and/or A-US to estimate GA or used calibrated newborn weighing scales for birthweight estimation were included. The risk of bias was assessed using the Quality Assessment of Diagnostic Accuracy Studies-II tool and evaluated the quality of the reference standards. When sufficient data were available, pooled estimates were calculated using random-effects models.

**Results:**

A total of 50 studies were included. A-US was a reasonable tool for GA assessment if conducted by physicians using fetal biometry and the Hadlock method for GA estimation. LMP was reasonable when women had regular cycles, knew their LMP, were not using contraceptives and LMP data were collected by healthcare providers. When A-US was used as the reference standard, PCS methods estimated GA with a precision of ±2.8 to ±3.2 weeks. FL measurement <7.5 cm showed a pooled sensitivity of 76.2% and specificity of 36.6% for identifying preterm birth. FL measurement ≤7.6 cm had a pooled sensitivity of 78.6% and specificity of 65.7% for identifying low birth weight (LBW). High heterogeneity across studies was observed.

**Conclusion:**

This systematic review and meta-analysis highlights significant variability and methodological inconsistencies in using PCS methods and FL measurement for estimating GA and LBW in LMICs. The observed high heterogeneity across studies suggests a cautious interpretation of the results.

**PROSPERO registration number:**

CRD42020209455.

**WHAT IS ALREADY KNOWN ON THIS TOPIC**

- One in five newborns in low-income and middle-income countries (LMICs) is born prematurely or with low birth weight (LBW), increasing their susceptibility to neonatal mortality. Early detection and intervention for these infants can be life-saving.
- Postnatal clinical scoring (PCS) methods and foot length (FL) measurements are commonly used to estimate gestational age (GA) and LBW in LMICs.

**WHAT THIS STUDY ADDS**

- PCS methods such as Ballard Score and Dubowitz Score tend to overestimate GA while the Eregie scoring model underestimates it due to high variability across the studies.
- The diagnostic accuracy of FL measurements for prematurity and LBW shows varying sensitivity and specificity due to significant methodological differences and high heterogeneity across studies.

**HOW THIS STUDY MIGHT AFFECT RESEARCH, PRACTICE OR POLICY**

- There is an urgent need for standardised GA and birthweight measurement protocols, as well as consensus on reference standards, to improve the reliability and accuracy of PCS and FL assessments in LMICs.
- Enhancing these diagnostic tools will lead to better clinical decision-making and improved neonatal outcomes, particularly in diverse and resource-limited healthcare settings.
- Policies should prioritise skill development, quality assurance and supportive supervision for healthcare providers conducting GA and birthweight assessments.

## Introduction

 Preterm and low birth weight (LBW) pose significant challenges to neonatal health globally, particularly in low-income and middle-income countries (LMICs).^1 2^ In 2020, an estimated 13.4 million babies were born preterm,^1^ and 19.8 million were born with a birth weight <2500 g—LBW, globally.^2^ Approximately 900 000 preterm newborns die before the age of 5, with the majority of deaths occurring within the first week after birth, particularly in south Asia and sub-Saharan Africa.^3^ LBW increases the risk of neonatal mortality by nearly 20 times compared with normal-weighted infants.^4^ The causes of death due to preterm birth and LBW are often preventable, emphasising the importance of early detection and prompt management.^5^

Antenatal ultrasound (A-US) is the gold-standard method for estimating gestational age (GA).^6 7^ However, its use in LMICs is limited due to factors such as limited availability, inadequate maintenance of US devices, late presentation of pregnant women for antenatal care (ANC) and high cost.^8^,^10^ In settings where access to A-US is limited, the last menstrual period (LMP) is often used to estimate GA, but this method is prone to errors due to inaccurate recall or irregular menstrual cycles or women on contraception 3 months prior to conception or breastfeed at the time of conception.^11^ Postnatal clinical scoring (PCS) methods and foot length (FL) measurements have been established to identify preterm birth newborns and LBW.^12^ The Ballard and Dubowitz scores (DS) assess GA via physical and neurological newborn examinations,^12 13^ and the Eregie scoring model (ESM) determines newborn maturation using physical examination and anthropometric measurements.^12^ Anthropometric measurements such as mid-upper arm circumference, head and chest circumference and FL were tested to identify preterm and LBW. For this review, we selected FL measurement due to its simplicity, which makes it feasible for scaling up. FL measurement can be performed with locally available, low-cost tools such as a rigid transparent ruler, and it can be done with minimal handling of the baby.^14^

Two high-quality systematic reviews and meta-analyses, published in 2016^12^ on neonatal clinical examination including BS, DS, ESM and other methods of GA assessment and in 2020^15^ on diagnostic accuracy of FL for identification of preterm and LBW, reported that low quality of studies and high heterogeneity were the major limitations for interpretation. Both reviews also recommended studies with high-quality A-US as reference standard. Additionally, the WHO has emphasised the need for additional research to discover simple, reliable and feasible methods for assessing GA and birth weight in LMICs.^16^

Therefore, the objectives of this study were (1) to update the existing systematic reviews and meta-analyses on the diagnostic accuracy of PCS and FL for GA and birthweight assessment in a single review in the LMIC context and (2) to assess the quality of evidence related to reference standards of (1) A-US, (2) LMP, (3) PCS and (4) newborn weighing scales.

## Materials and methods

This systematic review and meta-analysis was based on original studies building on a previous review that examined studies up to June 2022. The Preferred Reporting Items for Systematic Reviews and Meta-Analyses was used and is available as online supplemental material. The study was registered at the International Prospective Register of Systematic Reviews—PROSPERO CRD42020209455.

### Search strategy

Systematic literature searches were conducted using databases including PubMed (Medline), Web of Science, Cochrane Library, Cumulative Index to Nursing and Allied Health Literature (CINAHL) and Scopus. A librarian (KM) from Aga Khan University, Karachi Pakistan, performed the searches and were exported to EndNote (V.X9, Clarivate Analytics). In this review, ESM, DS and BS were denoted as PCS methods. Detailed search terms are available in online supplemental table 1.

### Inclusion criteria

Original studies written in the English language from LMICs, published between 1 January 2000 and 29 February 2024, were included. Studies reported live births and assessed the diagnostic accuracy of PCS and/or FL for determining GA and birth weight, as well as identifying prematurity and LBW were included. Studies using the LMP, A-US, PCS and/or a calibrated newborn weighing scale as reference standard were included. Additionally, studies that used PCS as the reference standard for FL for GA were also included. LMICs were selected due to the significant healthcare challenges in these regions, which have the highest rates of preterm births and LBW. By including studies from the year 2000 onwards, the review aimed to capture contemporary practices and diagnostic standards, reflecting the transition from reliance on LMP to more accurate and widely adopted methods such as A-US and calibrated newborn weighing scales.

Studies reported stillbirths as the study population, reported small for GA as the only outcome, involved children with chromosomal abnormalities or assessed GA on or after day 7 of birth were excluded. Additionally, studies that did not use A-US or LMP as reference standards for GA or did not employ calibrated newborn weighing scales as the reference standard for LBW assessment were excluded. Case reports/series, narrative/scoping reviews, editorials and published abstracts were also excluded.

### Case definition

According to the WHO, preterm birth is defined as the birth of a baby <37 weeks of gestation^17^ and LBW is defined as birth weight <2500 g.^18^

### Data review and extraction procedure

After removing duplicate studies from the EndNote library, two independent reviewers (MU and QA) screened titles and abstracts to identify full-text articles meeting eligibility criteria. We then read full-text articles meeting these criteria and extracted data, including study title, journal, publication year, country, study design, setting (hospital vs community), population characteristics, sampling strategy, sample size, methods of assessing GA, reference standards, descriptive data (preterm birth and LBW frequencies), and diagnostic accuracy and agreement estimates (correlation coefficient, mean difference, SD, diagnostic accuracy measures such as sensitivity, specificity, positive predictive value (PPV), negative predictive value (NPV) and Bland Altman’s limits of agreement (LOA)). We entered the data into MS Excel.

### Quality assessment of eligible studies

The risk of bias in individual studies was assessed using the Quality Assessment of Diagnostic Accuracy Studies (QUADAS-2) tool, which evaluates diagnostic studies in four domains: selection of participants, index test, reference standards and flow and timing. Each domain received a score from 0 to 1, indicating a low to high risk of bias. MU and QA independently evaluated methodological quality, resolving disagreements through mutual discussion. If a consensus was not reached, a third reviewer (SST) reviewed the article for the final decision. In addition to QUADAS-2, we assessed the quality of reference standards, such as A-US and LMP (online supplemental table 2).

### Additional calculations

Bland Altman’s LOAs were used to observe any bias in reporting the mean difference between the two compared methods as part of the included studies’ quality and reporting bias assessments. LOA was calculated if studies mentioned either the mean±SD of GA for both test and reference standard methods or the mean difference and SD of the mean difference.^19^

$$LOA=Mean\;difference\;\pm Z\;\frac{\propto }{2}\;\left(SD\right)\;$$

The 95% CI was calculated for sensitivity, specificity, PPV, NPV and area under the curves, where applicable.^20^

$$95\%\;CI=proportion\;\pm Z\;\frac{\propto }{2}\;\left(Standard\;error\;of\;proportion\right)\;$$

$$Standard\;error\;of\;proportion=\;\sqrt{\frac{P\left(1-p\right)}{n}}$$

### Standardised effect size: pooled variance

Reported mean differences were transformed into standardised mean differences to facilitate comparison across heterogeneous studies with varying characteristics. Pooled variances and SDs around the pooled estimates were calculated using the formula.^21^

$${\text{ Variance }}_{\text{pooled }}=\frac{\sum_{i=1}^{k}\left(n_{i}-1\right)S_{i}^{2}}{\sum_{i=1}^{k}\left(n_{i}-1\right)}$$

### Data analysis

Data were summarised and grouped in tables based on methods of GA determination and the reference standard. Data analysis was performed by using STATA V.17 (StataCorp). Meta-analysis was employed when two or more studies had appropriate data for pooled analysis. Individual study-level mean differences between the two GA assessment methods were pooled using the ‘meta esize’ command, providing the pooled mean difference and 95% CI. To account for heterogeneity within the data, a meta-analysis method employing the random effects model (REM) was used, which accommodates variability across studies beyond what would be expected by chance alone. Higgins’s I^²^ was used to quantify the degree of heterogeneity present in the pooled data. Correlation coefficients were pooled if studies reported a Pearson correlation (r) using the ‘metan’ command, providing descriptive summaries as median and range. Sensitivity and specificity were pooled using the ‘metandi’ command and reported all pooled effect sizes alongside their 95% CI. Forest plots for REM meta-analysis models were created using the ‘meta forestplot’ command.

## Results

**Figure 1:**
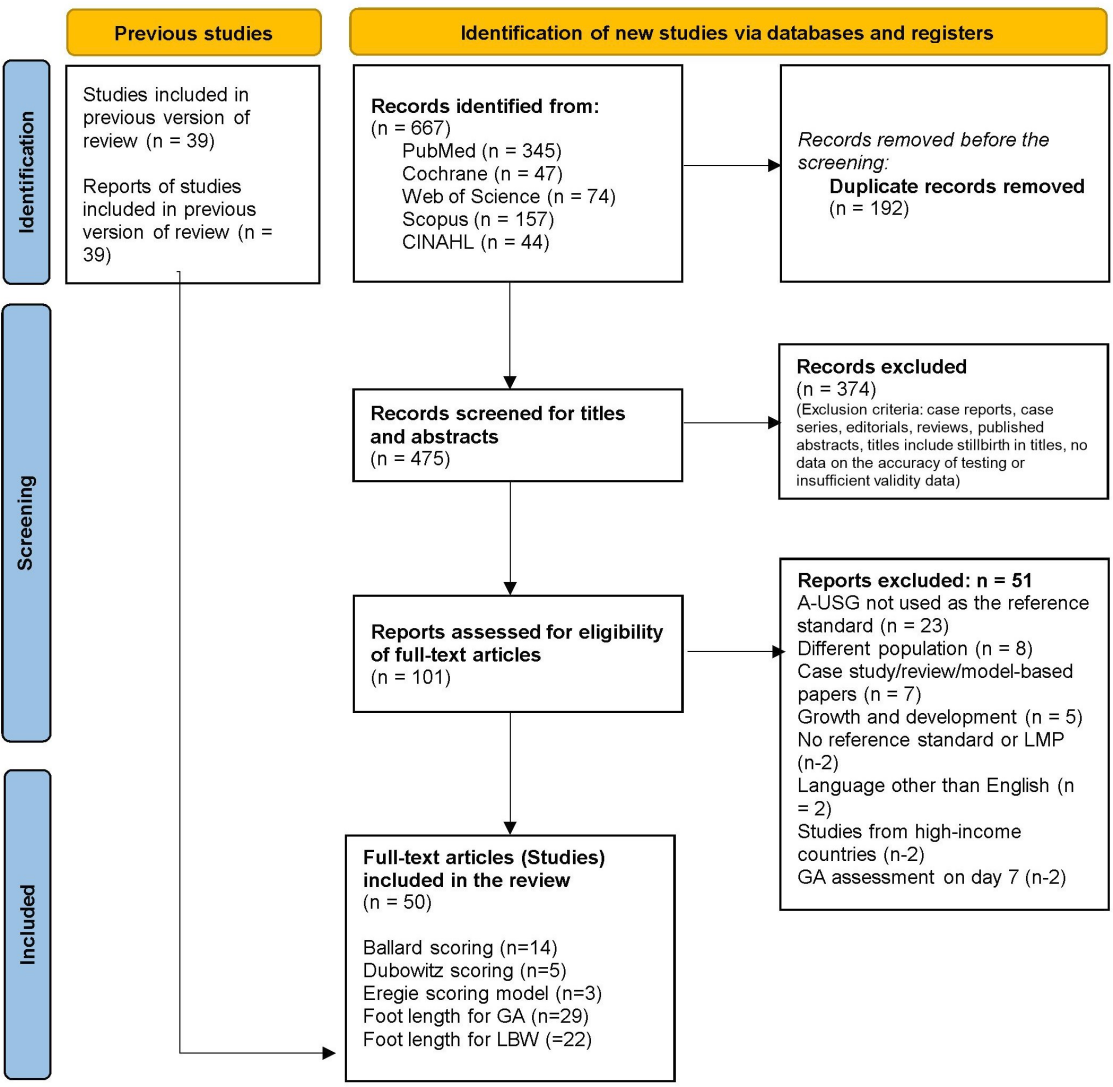
PRISMA flow diagram. GA, gestational age; LMP, last menstrual period; PRISMA, Preferred Reporting Items for Systematic Reviews and Meta-Analyses.

After a comprehensive search across all databases, 667 studies were identified. Following the removal of duplicates, 475 studies underwent screening for eligibility based on titles and abstracts. Subsequently, 101 full-text studies were identified for assessment regarding reporting criteria and reference standards. Ultimately, 50 studies were included in the systematic review (figure 1).

### Quality assessment

The QUADAS-2 summary graph indicated a high risk of bias related to patient selection and reference standards. Studies using LMP as a reference standard showed the high risk of bias attributed to recall bias. However, a low risk of bias was observed across other QUADAS-2 domains (online supplemental figure 1).

Characteristics of each study are summarised in online supplemental tables 2–7.

### Quality of the reference standards for GA and birth weight

#### A-US (n=18)

A-US was conducted by trained staff (n=10)^1022^,^30^ within 20 weeks of gestation (n=6)^24 26 27 29 31 32^ using portable US machines (n=6)^1012 27^,^29 33^ and fetal biometry (n=8)^10 12 22 25 27 29 34^ with the Hadlock method for GA estimation (n=5).^10 12 25 27 29^ Quality and reliability were assessed in seven studies^10 22 24 26 27 33 35^ (table 1).

**Table 1:** Quality assessment of reference standards. (A) Antenatal ultrasound, (B) Last menstrual period, (C) Postnatal clinical scoring and (D) Newborn weighing scales

(A) Antenatal ultrasound (n=18)								
Author, year	PCS/method	Setting	Who performed A-US	When was first A-US	Portable	Fetal biometry	Methods for GA	Quality/
								reliability
Taylor *et al*^22^ 2010	BS	Medical Research Council’s station	Community midwife	Second trimester	–	CRL and BPD	–	Yes
Wylie *et al*^10^ 2013	BS	Clinics	Physician	Second trimester	Portable	BPD, femur length, AC	Hadlock	Yes
Zahan *et al*^23^ 2017	BS	Hospital	Physician	–	–	–	–	–
Singhal *et al*^79^ 2017	BS	Hospital	–	First trimester	–	–	–	–
Unger *et al*^33^ 2019	BS	Hospital	–	Second and third trimester	Portable	CRL, femur length and BPD	–	Yes
AMANHI Study Group^35^ 2021	BS	Clinics	Trained staff	<20 weeks	Portable	CRL, femur length and BPD	Hadlock	Yes
Pietravalle *et al*^80^ 2022	BS	Hospital	–	Third trimester	–	–	–	–
Lee *et al*^24^ 2016	BS, ESM, FL	Community	Physician	<20 weeks	Portable	CRL and BPD	Hadlock	Yes
Stevenson *et al*^32^ 2021	BS, FL	Hospital	–	<13 weeks	–	–	–	–
Karunasekera *et al*^31^ 2002	DS	Hospital	–	15–20 weeks	–	–		–
Moore *et al*^25^ 2015	DS	Clinics	Sonographers	10–23 weeks	–	CRL and BPD	Robinson and Fleming, Altman and Chitty and Hadlock	–
Rosenberg *et al*^67^ 2009	DS, BS	Hospital	–	–	–	–	–	–
Raj *et al*^26^ 2021	ESM, BS	Hospital	Physician	<13 weeks	–	–	–	Yes
Wyk and Smith^30^ 2016	FL	Hospital	Sonographer students	<23 weeks	–	–	–	–
Paulsen *et al*^34^ 2019	FL	Community	–	First trimester	High resolution	CRL and BPD	–	–
Tregstina *et al*^66^ 2021	FL	Hospital	–	First trimester	–	–	–	–
Mengi *et al*^28^ 2023	FL	Hospital	Midwives	First ANC visit	Portable	–	–	–
Tikmani *et al*^27^ 2024	FL	Community	Sonographers	<20 weeks	Portable	CRL, BPD, femur length	Hadlock	Yes

(B) Last menstrual period (n=11)											
Author, year	PCS/FL method	Setting	Who took LMP	Aware of LMP	Regular cycles	Not on contraception, 3 months before conception	BF before conception	Pregnancy complications	When LMP asked	Method of assessing GA	LMP Reliable
Zahan *et al*^23^ 2017	BS	Hospital	–	Yes	–	–	–	–	–	–	Yes
Rada *et al*^41^ 2018	BS	Community	–	Yes	–	–	–	–	–	–	
Sunjoh *et al*^38^ 2004	BS, DS, ESM	Hospital	–	Yes	Yes	Yes	Yes	Yes	–	–	–
Feresu *et al*^43^ 2002	DS, BS	Hospital	Nurse/one trained staff	Yes	Yes	Yes	–	–	20–24 weeks	–	Yes
Thawani *et al*^37^ 2013	FL	Hospital	–	Yes	Yes	Yes	Yes	–	First trimester	Naegele’s formula	–
Singhal *et al*^39^ 2014	FL	Hospital	–	Yes	–	–	–	–	–	Naegele’s formula	–
Kc, *et al*^65^ 2015	FL	Hospital		Yes	–	–	–	–		–	–
Pratinidhi *et al*^42^ 2017	FL	Hospital	–	Yes	–	–	–	–	–	–	–
Tiruneh^36^ 2020	FL	Hospital	Midwives	Yes	Yes	–	–	Yes		Naegele’s formula	–
Dereje *et al*^44^ 2023	FL	Hospital	–	Yes	Yes	–	–	–	Early trimester	Naegele’s formula	–
Sintayehu *et al*^40^ 2023	FL	Hospital	–	Yes	–	–	–	–	–	–	–

(C) Postnatal clinical scoring (n=17)						
Author, year	Method	Description of PCS	When performed after birth	Who did test method	Methods to control bias in test method	Remarks
PCS- (Ballard scoring)						
Mukherjee *et al*^59^ 2013	FL	–	–	–	–	Just mentioned—BS used to assess GA
Singhal *et al*^39^ 2014	FL	–	–	–	–	Just mentioned—BS used to assess GA
Gavhane *et al*^52^ 2016	FL	–	1 day	–	–	Just mentioned—BS used to assess GA
Srivastava *et al*^46^ 2015	FL	*Partially described	24 hours	–	–	–
Thi *et al*^49^ 2015	FL	†Well described	24 hours	Paediatrician	–	–
Wyk and Smith^30^ 2016	FL	–	24 hours	–	–	Just mentioned—BS used to assess GA
Srinivasa *et al*^45^ 2017	FL	*Partially described	48 hours	–	–	–
Roy *et al*^69^ 2019	FL	–	–	–	–	Just mentioned—BS used to assess GA
Tenali and Tenali^50^ 2019	FL	–	1 day	–	–	Just mentioned—BS used to assess GA
Gidi *et al*^48^ 2020	FL	†Well described	–	Paediatrician	–	–
Kapoor and Soni^51^ 2020	FL	–	24 hours	Paediatric resident	–	–
Dagnew *et al*^54^ 2020	FL	–	–	Paediatric resident	–	Just mentioned—BS used to assess GA
Keshwani *et al*^68^ 2020	FL	–	–	–	–	Just mentioned—BS used to assess GA
Rafat *et al*^53^ 2020	FL	–	1 day	–	–	Just mentioned—BS used to assess GA
Srinavasa *et al*^47^ 2020	FL	*Partially described	48 hours	–	–	–
PCS - (ESM)						
Marchant *et al*^55^ 2010	FL	*Partially described	1 day	Clinical officers	–	–
Nabiwemba *et al*^56^ 2013	FL	*Partially described	–	Midwives	–	–
Gidi *et al*^48^ 2020	FL	†Well described	–	Paediatrician	–	–

(D) Newborn weighing scales (n=22)					
Author, year	When BW taken	Clothing status of baby	Type of scale	Calibration	Unit
Ahmed^57^ 2014	<24 hours	Naked	Digital	–	kg
Alia *et al*^58^ 2011	<24 hours	–	Not sure (paediatric weighing machine)	–	g
Gavhane *et al*^52^ 2016	<24 hours	–	Digital	–	kg
Mukherjee *et al*^59^ 2013	<24 hours	–	Digital	–	kg
Mullany *et al*^60^ 2007	<24 hours	–	Digital	Calibrated with bottle of 1000 gm	g
Pratinidhi *et al*^42^ 2017	<24 hours	Naked	Digital	–	kg
Rustagi *et al*^61^ 2012	<24 hours	Minimal clothing	Digital	–	g
Srinivasa *et al*^45^ 2017	<48 hours	Naked	Not sure (paediatric weighing machine)	–	Kg
Srivastava *et al*^46^ 2015	<24 hours	Naked	Digital	–	g
Thi *et al*^49^ 2015	<24 hours	–	Digital	Calibrated	g
Kc *et al*^65^	<24 hours	–	Manual	Calibrated	g
Tregstina *et al*^66^ 2021	<48 hours	–	Digital	–	g
Gidi *et al*^48^ 2020	<24 hours	–	Digital	Calibrated	g
Hadush *et al*^62^ 2017	<24 hours	Naked	Digital	Calibrated with bottle of 1000 gm	g
Marchant *et al*^55^ 2010	–	–	Digital	Calibrated with bottle of 1000 gm	g
Modibbo and Taura^63^ 2013	<24 hours	–	Digital	–	–
Nabiwemba *et al*^56^ 2013	<24 hours	–	Digital	Calibrated with bottle of 1000 g	g
Otupiri *et al*^64^ 2014	<24 hours	Naked	Digital	Calibrated with bottle of 1000 g	kg
Paulsen *et al*^34^ 2019	<24 hours	Naked	Digital	Calibrated	
Sintayehu *et al*^40^ 2023	<24 hours	–	Digital	Calibrated	g
Mengi *et al*^28^ 2023	<72 hours	–	Digital	Calibrated	kg

(–) mean information is not available.

* Partially described, means only a few clinical signs were mentioned.

† Well described, mean partially described plus scoring were mentioned.

ACabdominal circumferenceANCantenatal careA-USantenatal ultrasoundBFbreast feedingBPDbiparietal diameterBSBallard ScoreCRLcrown-rump lengthDSDubowitz ScoreESMEregie scoring modelFLfoot lengthGAgestational ageLMPlast menstrual periodPCSpostnatal clinical scoring

#### LMP (n=11)

All 11 studies included women who were aware of their LMP. Criteria for inclusion were women aware of their LMP (n=11),^2336^,^44^ regular menstrual cycles (n=5),^36^,^3843 44^ no contraception use in the 3 months prior to conception (n=3),^37 38 43^ no breast feeding after conception (n=2)^37 38^ and the absence of pregnancy complications (n-2).^36 38^ LMP data were collected by midwives or nurses (n=2)^36 43^ in the early trimester (n=2),^43 44^ with GA assessed through Naegele’s formula (n=4)^36 37 39 44^ and reliability assessed in two studies^23 43^ (table 1).

#### PCS (n=17)

15 studies validated FL against the Ballard Score (BS) as a reference standard, and three studies used the ESM as a reference standard. Of 15 studies, 5 studies described the procedures (partially described: only clinical signs, n=3^45^,^47^; well described: clinical signs and scoring, n=2^48 49^). BS was performed within 24 hours/1 day after birth (n=7)^3046 49^,^53^ by paediatricians (n=4).^48 49 51 54^ Three studies described the ESM, which was conducted on day 1 (n=1)^55^ by paediatricians (n=2)^48 55^ and midwives (n=1)^56^ (table 1).

#### Newborn weighing scales (n=22)

Newborns were measured within 24 hours of birth (n=17),^3440 42 48 49 52 56^,^65^ either naked or with minimal clothing (n=8)^34 42 45 46 57 61 62 64^ using digital (n=18)^2834 40 42 46 48 49 52 55^,^57 59^ calibrated weighing scales (n=11),^28 34 40 48 49 55 56 60 62 64 65^ with weights recorded in grams (n=12).^4043 46 48 49 55 58 60^,^62 65 66^ FL measurements were taken by averaging two or three readings recorded (n=6)^28 40 48 49 56 64^ (table 1).

### Diagnostic accuracy of PCS methods in assessing the GA

#### Ballard scoring with A-US as a reference standard (n=10)

In seven studies,^10 24 26 32 33 35 67^ BS resulted in a pooled mean difference of 0.65 weeks (95% CI −0.23 to 1.54, p<0.001) and a pooled SD of 1.6 weeks. Four out of 10 studies reported a correlation coefficient ranging from 0.31 to 0.94.^12 26 32 33^ Additionally, four studies^12 32 33 35^ reported a pooled sensitivity and specificity of 67.0% (95% CI 22.0% to 94.0%) and 80.0% (95% CI 73.0% to 85.0%), respectively, for identifying preterm births (table 2 and online supplemental figures 2–4).

**Table 2:** Validity of postnatal clinical scoring in assessing gestational age keeping antenatal ultrasound and last menstrual period as a reference standard

First author	Country	Clinical scoring	Study design	Sample size	Preterm (%)	Mean difference	Bland Altman (LOA)	Correlation	Sensitivity, %(95% CI)	Specificity, %(95% CI)
Antenatal ultrasound as reference standard										
Rosenberg *et al*^67^ 2009	Bangladesh	BS	Cohort study	355	–	2.9±7.8	(–4.9 to 10.6)			
Taylor *et al*^22^ 2010	Gambia	BS	Cohort study	80	25.0	–	–	0.70		
Wylie *et al*^10^ 2013	Malawi	BS	Cohort study	178	4.6	0.8±2.2	(–3.5 to 5.1)			
Lee *et al*^24^ 2016	Bangladesh	BS	Cohort study	1066	11.4	−0.4±2.4	(–4.7 to 4.0)		15.0(12.8 to 17.1)	87.0(85.0 to 89.0)
Zahan *et al*^23^ 2017	Bangladesh	BS	Cross-sectional study	129	–	–	–	0.94		
Unger *et al*^33^ 2019	*Multicountry study (Africa)	BS	Cohort study	1630	15.0	0.80	(–3.5 to 5.1)	0.31	42.0(39.6 to 44.4)	77.0(75.0 to 79.0)
Raj *et al*^26^ 2021	India	BS	Cross-sectional study	1114	8.8	0.65	(–0.9 to 2.3)			
Stevenson *et al*^32^ 2021	South Africa	BS	Prospective study	106	78.3	−0.14	(–2.9 to 2.7)	0.93	97.6(94.7 to 100)	73.9(65.5 to 82.3)
AMANHI Study Group^35^ 2021	†Multicountry study (Asia & Africa)	BS	Cohort study	7428	7.9	−1.96	(–15.3 to 33.6)		80.0(79.1 to 80.9)	80.0(79.1 to 80.1)
Pietravalle *et al*^80^ 2022	Tanzania	BS	Retrospective	70	–	1.2	(–1.8 to 4.2)	–	–	–
Karunsekera *et al*^31^ 2002	Sri Lanka	DS	Cross-sectional study	200	–	2.2±1.4	–	–	–	–
Rosenberg *et al*^67^ 2009	Bangladesh	DS	Cohort study	355	–	−3.6±3.6	(–11.0 to 3.3)	0.91		
Moore *et al*^25^ 2015	Thailand	DS	Longitudinal cohort	250	28.0	2.6±1.3	(0.5 to 4.6)	–	61.0(54.9 to 76.0)	99.0(97.7 to 100)
Lee *et al*^24^ 2016	Bangladesh	ESM	Study nested within-cluster randomised trial	1066	11.4	−2.0±1.6	(–5.4 to 1.5)	–	75.0(72.4 to 77.6)	58.0(55.0 to 60.9)
Raj *et al*^26^ 2021	India	ESM	Cross-sectional study	1114	8.8	0.3±0.9	(–1.4 to 2.1)	–		
Last menstrual period as reference standard										
Feresu *et al*^43^ 2002	Zimbabwe	BS	Cross-sectional study	364	23.9	–	–	0.80	–	–
Sunjoh *et al*^38^ 2004	Cameroon	BS	Cross-sectional study	358	31.8	0.36±1.5	(–2.6 to 3.3)	0.93	–	–
Zahan *et al*^23^ 2017	Bangladesh	BS	Cross-sectional study	129	–	–	–	0.94	–	–
Rada *et al*^41^ 2018	‡Multi-country study (Africa)	BS	Cohort study	4390	–	−1.24±6.3	(–13.6 to 11.1)	–	–	–
Feresu *et al*^43^ 2002	Zimbabwe	DS	Not available	364	23.9		–	0.81	–	–
Sunjoh *et al*^38^ 2004	Cameroon	DS	Cross-sectional study	358	31.8	0.50±1.3	–	0.94	–	–
Sunjoh *et al*^38^ 2004	Cameroon	ESM	Cross-sectional study	358	31.8	0.26±1.4	–	0.93	–	–

* Burkina Faso, Ghana, Malawi and Zambia.

† Bangladesh, Ghana, Pakistan, Tanzania, and Zambia.

‡ Benin, Gabon, Mozambique, and Tanzania.

BSBallard ScoreDSDubowitz ScoreESMEregie scoring modelLOAlimits of agreement

#### Ballard scoring with LMP as a reference standard (n=4)

BS resulted in a pooled mean difference of −0.35 weeks (95% CI −0.75 to 0.05, p=0.04) and a pooled SD of 1.5 weeks.^38 41 67^ Three studies reported a correlation coefficient of 0.94.^23^ None reported sensitivity and specificity (table 2).

#### Dubowitz scoring with A-US as a reference standard (n=3)

DS resulted in a pooled mean difference of 0.68 weeks (95% CI 0.52 to 0.84, p=0.35) and a pooled SD of 1.4 weeks.^25 67^ One out of two studies reported a Pearson correlation coefficient of 0.91. One study reported a sensitivity of 61.0% (95% CI 54.9% to 76.0%) and a specificity of 99.0% (95% CI 97.7% to 100%) for identifying preterm birth neonates.^25^ (table 2)

#### Dubowitz scoring with LMP as a reference standard, (n=2)

DS resulted in a pooled mean difference of 0.67 weeks (95% CI 0.45 to 0.89, p=0.52) and a pooled SD of 1.2 weeks.^31 38^ Two studies reported the Pearson correlation coefficient ranged from 0.81 to 0.94.^38 43^ None of the studies reported sensitivity and specificity. (table 2)

#### ESM with A-US as a reference standard (n=2)

ESM resulted in a pooled mean difference of −0.44 weeks (95% CI −0.51 to −0.37, p<0.001) and a pooled SD of 1.4 weeks.^24 26^ One study reported a sensitivity of 75.0% (95% CI 72.4% to 77.6%) and specificity of 58.0% (95% CI 55.0% to 60.9%) of ESM for identifying preterm birth neonates.^24^ (table 2)

#### Eregie scoring with the LMP as a reference standard (n=1)

One study reported Pearson correlation coefficient of 0.93 and a mean difference of 0.26±1.38 weeks.^38^ (table 2)

### Diagnostic accuracy of FL for GA assessment

#### The FL with A-US as a reference standard (n=7)

Three studies reported positive correlations between FL and GA ranging from 0.37 to 0.89 with a pooled correlation coefficient of 0.72 (95% CI 0.38 to 1.05).^30 34 40 44 66^ Two studies used an FL cut-off of <7.5 cm to detect preterm birth with FL measurements <7.5 cm (n=2) showed pooled sensitivity of 76.2 (95% CI 70.2 to 81.5) and pooled specificity of 36.6 (95% CI 32.7 to 40.7) for identifying preterm birth compared with A-US.^24 32^ table 3

**Table 3:** Comparison of foot length for estimating gestational age with A-US, LMP and postnatal clinical scoring (Ballard and Eregie score model) as reference standards

Study	Country	Study design	Sample size	Preterm (%)	Correlation coefficient	FL Cut-offs(cm)	Sensitivity, %(95% CI)	Specificity, %(95% CI)
A-US as the reference standards								
Lee *et al*^24^ 2016	Bangladesh	Cross-sectional study	710	8.3	–	<7.5	64.0(60.5 to 67.5)	35.0(31.5 to 38.5)
Wyk and Smith^30^ 2016	South Africa	Not specified	200	–	0.89	–	–	–
Paulsen *et al*^34^ 2019	Tanzania	Observational study	376	4.5	0.37	≤7.7	94.0(71.0 to 100)	64.0(59.0 to 69.0)
Stevenson *et al*^32^ 2021	South Africa	Observational study	106	78.3	–	<7.5	98.9(93.4 to 100)	60.9(38.5 to 80.3)
Tergestina *et al*^66^ 2021	India	Cross-sectional study	520	–	0.89	–	–	–
Mengi *et al*^28^ 2023	Papua New Guinea	Prospective study	342	7.3	–	<7.7	88.0(70.0 to 95.8)	61.8(56.5 to 67.0)
Tikmani *et al*^27^ 2024	Pakistan	Cross-sectional study	336	22.3	–	<7.6	90.8(86.6 to 97.6)	96.0(88.8 to 99.2)
LMP as the reference standards								
Thawani *et al*^37^ 2013	India	Cross-sectional study	1000	37.3	0.51	–	–	–
Singhal *et al*^39^ 2014	India	Observational study	1000	36.5	0.93	7.0	94.8(93.4 to 96.2)	94.3(93.0 to 95.7)
Kc *et al*^65^ 2015	Nepal	Cross-sectional study	811	6.7	–	7.5	32.7(29.5 to 35.9)	83.8(81.3 to 86.3)
Pratinidhi *et al*^42^ 2017	India	Not specified	645	6.7	0.63	<6.8	93.0(80.9 to 98.5)	86.7(83.7 to 89.3)
Tiruneh^36^ 2020	Ethiopia	Cross-sectional study	424	15.1	0.14	–	–	–
Sintayehu *et al*^40^ 2023	Ethiopia	Cross-sectional study	381	26.7	0.48	<7.1	77.0(75.2 to 80.4)	90.7(88.7 to 92.4)
Dereje *et al*^44^ 2023	Ethiopia	Cross-sectional study	420	16.4	0.46	–	–	–
Postnatal clinical scoring (Ballard scoring)								
Mukherjee *et al*^59^ 2013	India	Cross-sectional study	351	48.1	0.89	7.75	92.3	86.3
Singhal *et al*^39^ 2014	India	Observational study	1000	15.4	–	7.0	94.8	94.3
Gavhane *et al*^52^ 2016	India	Observational study	800	15.5	0.81	–	–	–
Srivastava *et al*^46^ 2015	India	Not specified	254	59.8	0.96	–	–	–
Thi *et al*^49^ 2015	Vietnam	Observational study	485	49.0	–	7.3	80.0(74.0 to 85.0)	81(76.0 to 86.0)
Wyk and Smith^30^ 2016	South Africa	Not specified	200	–	0.88	–	–	–
Srinivasa *et al*^45^ 2017	India	Cross-sectional study	500	16.8	0.86	7.4	98.8(93.5 to 100)	79.1(74.9 to 82.9)
Roy *et al*^69^ 2019	India	Cross-sectional study	320	17.5	–	7.35	80.0	78.0
Tenali and Tenali^50^ 2019	India	Prospective study	300	28.0	0.79	–	–	–
Gidi *et al*^48^ 2020	Ethiopia	Cross-sectional study	1389	10.2	–	7.5	81.7(74.3 to 87.7)	77 .0(74.6 to 79.3)
Kapoor and Soni^51^	India	Cross-sectional study	514	28.4	0.80	6.83	94.6	42
Dagnew *et al*^54^ 2020	Ethiopia	Cross-sectional study	–	33.2	0.87	7.35	98.5(92.1 to 99.7)	96.3(91.7 to 98.4)
Keshwani and Suroshe^68^ 2020	India	Cross-sectional study	350	–	0.78	–	–	–
Rafat *et al*^53^ 2020	Egypt	Prospective study	1000	–	0.69	–	–	–
Srinavasa *et al*^47^ 2020	India	Cross-sectional study	173	29.5	0.91	–	–	–
Postnatal clinical scoring - (Eregie scoring model)								
Marchant *et al*^55^ 2010	Tanzania	Cross-sectional study	529	9.0	–	<8.0	93.0(82.0 to 99.0)	58.0(53.0 to 62.0)
Nabiwemba *et al*^56^ 2013	Uganda	Cross-sectional study	711	4.0	0.76	7.5	85.7	90.4
Gidi *et al*^48^ 2020	Ethiopia	Cross-sectional study	1389	10.2	–	≤7.4	80.5(69.9 to 88.7)	91.4(89.7 to 92.8)

A-USantenatal ultrasoundLMPlast menstrual period

#### The FL with the LMP as a reference standard (n=7)

Six studies reported positive correlations between FL and GA ranging from 0.14 to 0.93 with a pooled correlation coefficient of 0.56 (95% CI 0.24 to 0.88).^36 37 39 42^ One study reported FL cut-off of <7.5 cm to identify preterm birth, with sensitivity of 32.7% and specificity of 83.8%. (table 3)

#### The FL with PCS as reference standard (n=17)

BS (n=15),^3940 45^,^47 49^ ESM (n=2)^55 56^ and both BS and ESM (n=1)^48^ were used as reference standards. 11 studies reported correlations ranging from 0.69 to 0.96.^3045^,^47 50^ One study reported FL cut-off of <7.5 cm to identify preterm birth using BS as reference standard, has sensitivity of 81.7% and specificity of 77%^48^ and another study used ESM as reference standard reported sensitivity of 85.7% and specificity of 90.4% for identification of preterm.^56^ (table 3)

### Diagnostic accuracy of FL in assessing the LBW

Of 22 studies that reported diagnostic accuracy of FL for identification of LBW, 15 studies showed a correlation ranging from 0.21 to 0.97 between FL and birth weight with a pooled correlation coefficient of 0.71 (95% CI 0.60 to 0.82).^3440 45 46 49 52 56 58 61^,^64 66 70^ Pooled sensitivity and specificity for identifying LBW at an FL cut-off of ≤7.4 cm (n=4) were 72.1 (95% CI 68.3 to 75.7) and 84.9 (95% CI 83.2 to 86.5), respectively. At an FL cut-off of ≤7.6 cm (n-4), the pooled sensitivity and specificity were 78.6 (95% CI 73.7 to 83.6) and 65.7 (95% CI 63.3 to 68.1), respectively.^28 34 48 61^ (table 4)

**Table 4:** Comparison of diagnostic accuracy of foot length in predicting low birth weight (LBW)

First author and year	Country	Study design	Sample size	LBW (%)	Correlation	Foot length cut-offs (cm)	Sensitivity, % (95% CI)	Specificity, % (95% CI)
Mullany^60^ 2007	Nepal	Not specified	1640	28.6	–	7.5	97.4(96.6 to 98.2)	32.7(30.4 to 35.0)
Marchant^55^ 2010	Tanzania	Cross-sectional study	529	15.0	–	<8.0	87.0(79.0 to 94.0)	60.0(55.0 to 64.0)
Alia^58^ 2011	Bangladesh	Cross-sectional study	100	52.0	0.77	–	–	–
Rustagi^61^ 2012	India	Prospective observational study	283	–	0.21	≤7.7	58.0(52.2 to 63.8)	83.0(78.6 to 87.4)
Mukherjee^59^ 2013	India	Cross-sectional study	351	51.8	0.95	<7.85	100	95.3(93.1 to 97.5)
Nabiwemba^56^ 2013	Uganda	Cross-sectional study	706	12.0	0.76	<7.9	94.1(86.8 to 98.1)	82.6(79.8 to 86.1)
Modibbo^63^ 2013	Nigeria	Cross-sectional study	551	–	0.66	–	–	–
Otupiri^64^ 2014	Ghana	Cross-sectional study	973	21.7	0.53	≤7.4	–	–
Ahmed^57^ 2014	India	–	1028	–	0.51	7.8	90.9(89.1 to 92.7)	33.3(30.4 to 36.2)
Thi^49^ 2015	Vietnam	Prospective observational study	485	51.0	–	≤7.4	85.0(70.0 to 89.0)	86.0(81.0 to 90.0)
Kc^65^ 2015	Nepal	Cross-sectional study	811	3.7	–	7.5	82.2(79.6 to 84.8)	85.2(82.8 to 87.6)
Srivastava^46^ 2015	India	–	254	–	0.97	–	–	–
Gavhane^52^ 2016	India	Prospective observational study	800	25.5	0.49	–	–	–
Hadush^62^ 2017	Ethiopia	Cross-sectional study	422	27.0	0.75	7.35	72.8(68.6 to 77.0)	91.6(89.0 to 94.2)
Srinivasa^45^ 2017	India	Cross-sectional study	500	–	0.90	<7.4	97.0(95.5 to 98.5)	87.1(84.2 to 90.0)
Pratinidhi^42^ 2017	India	–	645	–	0.75	–	–	–
Paulsen^34^ 2019	Tanzania	Prospective observational study	376	10.5	0.66	≤7.7	74.0(61.0 to 83.0)	67.0(61.0 to 72.0)
Gidi^48^ 2020	Ethiopia	Cross-sectional study	1486	13.7	–	≤7.7	84.2(78.4 to 88.9)	73.9(71.3 to 6.4)
Tregstina^66^ 2021	India	Cross-sectional study	520	–	0.97	–	–	–
Mengi^28^ 2023	Papua New Guinea	Prospective study	342	7.3	–	<7.7	84.7(74.7 to 91.2)	69.6(63.9 to 4.8)
Sintayehu^40^ 2023	Ethiopia	Cross-sectional study	381	26.7	0.53	<6.9	94.8(93.2 to 96.1)	80.5(77.9 to 82.9)

## Discussion

Existing reviews on PCS and FL as methods for GA and birthweight assessment within the context of LMICs have been updated. PCS methods such as BS and DS tend to overestimate GA while ESM underestimates it. Additionally, studies investigating the diagnostic accuracy of FL as a proxy for prematurity or LBW showed varying degrees of sensitivity and specificity; however, due to high heterogeneity, one should interpret these results with caution (online supplemental figures 2–4). The significant methodological differences, especially in the standardisation of reference standards like A-US and LMP, largely account for the observed variation and equivocal findings in existing studies on PCS methods and FL measurements.

Several contextual factors contribute to this variability. Many studies relied on secondary data lacking standardised data collection methods, leading to inconsistent findings. Methodological differences included anatomical landmarks and measurement tools for FL, with studies using landmarks such as heel to hallux or longest toe and tools ranging from plastic rulers to callipers to flexible tapes. Various cut-offs for FL as a proxy for prematurity (7.1–7.9 cm) and LBW (<6.9 to <7.9 cm) also led to differences in sensitivity and specificity.^28 34 71^ This variation in cut-offs may be attributed to the higher frequency of premature newborns in some studies.^40 56^ Furthermore, most studies evaluating FL and PCS for GA were conducted in hospital settings. Hospital settings, with higher incidences of prematurity, asphyxia, sepsis, growth retardation and maternal complications such as pre-eclampsia/eclampsia, gestational diabetes and anaemia, further added to this variability.^72 73^ Moreover, differences in healthcare settings, staff training and access to care between urban hospitals and rural areas in LMICs also contributed to this heterogeneity. Hospital-based studies often involve trained medical personnel, whereas rural settings may lack such resources, impacting the accuracy and generalisability of PCS and FL measurements.

South Asia exhibits a high prevalence of premature and LBW neonates, with variations in the diagnostic accuracy and optimal cut-offs for FL measurements when compared with other regions such as sub-Saharan Africa. Studies conducted in Asia show FL cut-offs ranging from <6.8^42^ to <7.75 cm^59^ for identifying preterm, whereas studies from Africa have cut-offs ranging from <7.1^40^ to <8 cm^74^ for the same purpose. Similarly, for identifying LBW neonates, Asian studies report FL cut-offs ranging from <7.4^45 49^ to <8.0 cm^59^ while African studies show cut-offs ranging from <6.9^40^ to <8.0 cm.^55^ These differences arise due to distinct population characteristics and genetic profiles, necessitating different cut-offs.^75 76^ This regional variability highlights the inherent complexity of applying a one-size-fits-all approach to neonatal assessments.^77^ Universal application without adjustments can lead to inaccurate assessments, potentially compromising the quality of care and intervention strategies. Therefore, while these diagnostic tools are valuable, their use must be tailored to regional contexts to achieve precise and reliable outcomes.^78^

This systematic review and meta-analysis has several limitations. First, relying on binary outcomes. Using categorical outcomes like LBW versus not LBW or preterm versus not preterm in LMICs offers advantages. These endpoints simplify data collection and interpretation, making it more feasible in resource-limited settings. Different cut-offs for continuous variables like FL introduce variability, complicating comparisons. Categorical outcomes provide clear, standardised criteria that facilitate decision-making and policy implementation and second, pooling individual-level data for continuous analysis was challenging due to logistical constraints, variations in data quality and limited access to advanced statistical tools, making categorical outcomes a more straightforward, actionable and accessible approach to addressing public health concerns in LMICs.

A deviation from the PROSPERO protocol in the manuscript regarding the inclusion of quality assessment of reference standards is acknowledged, as these factors contribute to heterogeneity across the studies. However, the overall methodology remains consistent with the PROSPERO protocol.

This study highlights the need for standardised measurement protocols and improved data collection methods. By carefully examining the quality of evidence related to reference standards, we recommend implementing uniform protocols for PCS and FL measurements across LMICs to ensure consistency and reliability. Additionally, it is crucial to invest in robust data collection and management systems to enhance the accuracy and applicability of GA and birthweight assessments. Furthermore, the policies should prioritise skill development, quality assurance and supportive supervision for healthcare providers conducting GA and birthweight assessments. These measures will ultimately lead to better neonatal health outcomes.

## Conclusion

In conclusion, this review reveals significant variability and methodological inconsistencies in using PCS methods and FL measurements for estimating GA and LBW in LMICs. The observed high heterogeneity across studies suggests a cautious interpretation of the results and calls for future research to be focused on validating and adapting these tools to better suit the specific contexts of diverse LMIC settings.

## supplementary material

10.1136/bmjpo-2024-002717 online supplemental file 1

## Data Availability

All data relevant to the study are included in the article or uploaded as online supplemental information.
