# Supplementary material for: Assessing the diagnostic accuracy of postnatal clinical scoring methods and foot length measurement for estimating gestational age and birthweight of newborns in low- and middle-income countries: a systematic review and meta-analysis
Source: BMJ Paediatr Open. 2024 Aug 30;8(1):e002717. doi: 10.1136/bmjpo-2024-002717 (PMC11367336; doi:10.1136/bmjpo-2024-002717)

6/24/2024

Supplementary material

Assessing the Diagnostic Accuracy of Postnatal Clinical Scoring Methods and Foot Length measurements for estimating gestational age and birthweight of newborns in Low- and Middle-Income Countries: A systematic review and meta-analysis

Supplementary materials

DEPARTMENT OF WOMEN'S AND CHILDREN'S HEALTH, UPPSALA UNIVERSITET, SWEDEN

Table 1: Search results

Search Engine	Search terms	# of hits per database
**Ballard Scoring**		
PubMed	("gestational age" [Mesh] OR "gestational age"[All Fields] OR "premature birth"[MeSH Terms] OR "premature birth"[All Fields] OR preterm[All Fields] OR "premature"[All Fields] OR prematurity[All Fields] OR ptb[All Fields] OR "fetal growth retardation"[MeSH Terms] OR "fetal growth restriction"[All Fields] OR "foetal growth restriction"[All Fields] OR "fetal growth retardation"[All Fields] OR "foetal growth retardation"[All Fields] OR "infant, low birth weight"[MeSH Terms] OR "low birth weight"[All Fields] OR "IUGR"[All Fields] OR "intrauterine growth restriction"[All Fields] OR "intrauterine growth retardation"[All Fields] OR "lbw"[All Fields] OR "birth weight"[MeSH Terms] OR "birth weight"[All Fields] OR "birthweight"[All Fields] OR "menstrual age"[All Fields] OR "fetal age"[All Fields] OR "foetal age"[All Fields] OR "fetal growth"[All Fields] OR "foetal growth"[All Fields] OR "embryo growth"[All Fields] OR "fetal development"[All Fields] OR "foetal development"[All Fields] OR infant[MeSH] OR neonatal[Mesh]) AND (“ballard score”[All Fields] OR “ballard examination”[All Fields] OR ”ballard exam”[All Fields])	44
Cochrane	(“gestational age” OR “premature birth” OR “preterm” OR “ptb” OR “fetal growth retardation” OR “fetal growth restriction” OR “intrauterine growth retardation” OR “intrauterine growth restriction” OR “low birth weight” OR “IUGR” OR “lbw” OR “birth weight”) AND (“ballard score” OR “ballard examination” OR ”ballard exam”)	36
Web of science	("gestational age" OR "premature birth" OR preterm OR "premature" OR prematurity OR "fetal growth retardation" OR "fetal growth restriction" OR "foetal growth restriction" OR "foetal growth retardation" OR "low birth weight" OR "IUGR" OR "intrauterine growth restriction" OR "intrauterine growth retardation" OR "lbw" OR "birth weight" OR "birthweight" OR "menstrual age" OR "fetal age" OR "foetal age" OR "fetal growth" OR "foetal growth" OR "embryo growth" OR "fetal development" OR "foetal development" OR infant OR neonatal) AND ("Validity" OR "Accuracy" OR "diagnostic" OR "Agreement") AND (“ballard score” OR “ballard examination” OR ”ballard exam”)	26
CINAHL	(Ballard score OR Ballard exam OR Ballard) AND (Preterm OR Prematurity OR Premature) AND (Accuracy OR correlation OR validity)	20
Scopus	TITLE-ABS-KEY (("gestational age" OR "premature birth" OR preterm OR "premature" OR prematurity OR "fetal growth retardation" OR "fetal growth restriction" OR "foetal growth restriction" OR "foetal growth retardation" OR "low birth weight" OR "IUGR" OR "intrauterine growth restriction" OR "intrauterine growth retardation" OR "lbw" OR "birth weight" OR "birthweight" OR "menstrual age" OR "fetal age" OR "foetal age" OR "fetal growth" OR "foetal growth" OR "embryo growth" OR "fetal development" OR "foetal development" OR infant OR neonatal) AND ("Validity" OR "Accuracy" OR "diagnostic" OR "Agreement") AND ("ballard score" OR "ballard examination" OR "ballard exam"))	32
**Dubowitz Score/exam**		
PubMed	("gestational age" [Mesh] OR "gestational age"[All Fields] OR "premature birth"[MeSH Terms] OR "premature birth"[All Fields] OR preterm[All Fields] OR "premature"[All Fields] OR prematurity[All Fields] OR ptb[All Fields] OR "fetal growth retardation"[MeSH Terms] OR "fetal growth restriction"[All Fields] OR "foetal growth restriction"[All Fields] OR "fetal growth retardation"[All Fields] OR "foetal growth retardation"[All Fields] OR "infant, low birth weight"[MeSH Terms] OR "low birth weight"[All Fields] OR "IUGR"[All Fields] OR "intrauterine growth restriction"[All Fields] OR "intrauterine growth retardation"[All Fields] OR "lbw"[All Fields] OR "birth weight"[MeSH Terms] OR "birth weight"[All Fields] OR "birthweight"[All Fields] OR "menstrual age"[All Fields] OR "fetal age"[All Fields] OR "foetal age"[All Fields] OR "fetal growth"[All Fields] OR "foetal growth"[All Fields] OR "embryo growth"[All Fields] OR "fetal development"[All Fields] OR	11

	"foetal development"[All Fields] OR infant[MeSH] OR neonatal[Mesh]) AND ("dubowitz score"[All Fields] OR "dubowitz examination"[All Fields] OR "dubowitz exam"[All Fields])	
Cochrane	(“gestational age” OR “premature birth” OR “preterm” OR “ptb” OR “fetal growth retardation” OR “fetal growth restriction” OR “intrauterine growth retardation” OR “intrauterine growth restriction” OR “low birth weight” OR “IUGR” OR “lbw” OR “birth weight”) AND (“dubowitz score” OR “dubowitz examination” OR “dubowitz method”)	6
Web of science	("gestational age" OR "premature birth" OR preterm OR "premature" OR prematurity OR "fetal growth retardation" OR "fetal growth restriction" OR "foetal growth restriction" OR "foetal growth retardation" OR "low birth weight" OR "IUGR" OR "intrauterine growth restriction" OR "intrauterine growth retardation" OR "lbw" OR "birth weight" OR "birthweight" OR "menstrual age" OR "fetal age" OR "foetal age" OR "fetal growth" OR "foetal growth" OR "embryo growth" OR "fetal development" OR "foetal development" OR infant OR neonatal) AND ("dubowitz score" OR "dubowitz examination" OR "dubowitz method") AND ("validity" OR "accuracy" OR "diagnostic" OR "agreement" OR "correlation")	7
CINAHL	gestational age AND Dobuwitz	5
Scopus	(("gestational age" OR "premature birth" OR "preterm" OR "ptb" OR "fetal growth retardation" OR "fetal growth restriction" OR "intrauterine growth retardation" OR "intrauterine growth restriction" OR "low birth weight" OR "IUGR" OR "lbw" OR "birth weight") AND ("dubowitz score" OR "dubowitz examination" OR "dubowitz method"))	43
**Eregie model**		
PubMed	(“gestational age”[MeSH Terms] OR “gestational age”[Text Word] OR “premature birth”[MeSH Terms] OR “premature birth”[Text Word] OR preterm[Text Word] OR ptb[Text Word] OR “fetal growth retardation”[MeSH Terms] OR “fetal growth restriction”[Text Word] OR “fetal growth retardation”[Text Word] OR “infant, low birth weight”[MeSH Terms] OR “low birth weight”[All Fields] OR “IUGR”[Text Word] OR “lbw”[Text Word] OR “birth weight”[MeSHTerms] OR “infant, low birth eight”[MeSH Terms]) AND “eregie”[All Fields]	9
Cochrane	(“gestational age” OR “premature birth” OR “preterm” OR “ptb” OR “fetal growth retardation” OR “fetal growth restriction” OR “intrauterine growth retardation” OR “intrauterine growth restriction” OR “low birth weight” OR “IUGR” OR “lbw” OR “birth weight”) AND (“eregie”)	0
Web of science	(“gestational age” OR “premature birth” OR “preterm” OR “ptb” OR “fetal growth retardation” OR “fetal growth restriction” OR “intrauterine growth retardation” OR “intrauterine growth restriction” OR “low birth weight” OR “IUGR” OR “lbw” OR “birth weight”) AND (“eregie”) AND ("validity" OR "accuracy" OR "diagnostic" OR "agreement" OR "correlation")	6
CINAHL	gestational age AND eregie	4
Scopus	(("gestational age" OR "premature birth" OR "preterm" OR "ptb" OR "fetal growth retardation" OR "fetal growth restriction" OR "intrauterine growth retardation" OR "intrauterine growth restriction" OR "low birth weight" OR "IUGR" OR "lbw" OR "birth weight") AND ("eregie"))	5
**Foot length**	*Ref: Diagnostic accuracy of neonatal foot length to identify preterm and low birth weight infants: a systematic review and meta-analysis*	

PubMed	"gestational age" [Mesh] OR "gestational age"[All Fields] OR "premature birth"[MeSH Terms] OR "premature birth"[All Fields] OR preterm[All Fields] OR "premature"[All Fields] OR prematurity[All Fields] OR ptb[All Fields] OR "fetal growth retardation"[MeSH Terms] OR "fetal growth restriction"[All Fields] OR "foetal growth restriction"[All Fields] OR "fetal growth retardation"[All Fields] OR "foetal growth retardation"[All Fields] OR "infant, low birth weight"[MeSH Terms] OR "low birth weight"[All Fields] OR "IUGR"[All Fields] OR "intrauterine growth restriction"[All Fields] OR "intrauterine growth retardation"[All Fields] OR "lbw"[All Fields] OR "birth weight"[MeSH Terms] OR "birth weight"[All Fields] OR "birthweight"[All Fields] OR "menstrual age"[All Fields] OR "fetal age"[All Fields] OR "foetal age"[All Fields] OR "fetal growth"[All Fields] OR "foetal growth"[All Fields] OR "embryo growth"[All Fields] OR "fetal development"[All Fields] OR "foetal development"[All Fields] OR infant[MeSH] OR neonatal[Mesh]) AND ("foot length"[All Fields] OR "foot size"[All Fields] OR "foot measurement"[All Fields] OR "footlength"[All Fields] OR ("foot"[All Fields] AND anthropometr*[All Fields]) OR "foot measure"[All Fields] AND ("validity" [All Fields] OR "diagnostic"[All fields] OR "Accuracy" [All Fields] OR "Agreement"[All Fields])	281
Cochrane	(“gestational age” OR “premature birth” OR “preterm” OR “ptb” OR “fetal growth retardation” OR “fetal growth restriction” OR “intrauterine growth retardation” OR “intrauterine growth restriction” OR “low birth weight” OR “IUGR” OR “lbw” OR “birth weight”) AND (foot length OR "foot size" OR "foot measurement" OR "footlength") AND ("valid" OR "Accuracy" OR "agreement" OR "Correlation")	5
Web of science	("gestational age" OR "premature birth" OR preterm OR "premature" OR prematurity OR "fetal growth retardation" OR "fetal growth restriction" OR "foetal growth restriction" OR "foetal growth retardation" OR "low birth weight" OR "IUGR" OR "intrauterine growth restriction" OR "intrauterine growth retardation" OR "lbw" OR "birth weight" OR "birthweight" OR "menstrual age" OR "fetal age" OR "foetal age" OR "fetal growth" OR "foetal growth" OR "embryo growth" OR "fetal development" OR "foetal development" OR infant OR neonatal) AND ("foot length" OR "foot size" OR "foot measurement" OR "foot length" OR ("foot" AND anthropometr*) OR "foot measure") AND ("Validity" OR "Accuracy" OR "diagnostic" OR "Agreement")	35
CINAHL	gestational age AND foot length AND (preterm OR birth weight OR low birth weight)	15
Scopus	(("gestational age" OR "premature birth" OR "preterm" OR "ptb" OR "fetal growth retardation" OR "fetal growth restriction" OR "intrauterine growth retardation" OR "intrauterine growth restriction" OR "low birth weight" OR "IUGR" OR "lbw" OR "birth weight") AND (foot AND length OR "foot size" OR "foot measurement" OR "footlength") AND ("valid" OR "Accuracy" OR "agreement" OR "Correlation"))	77

Table 2: Variables extracted from the individual studies for quality of antenatal ultrasound and last menstrual period

Antenatal Ultrasound	Last menstrual period
-Who performed A-US?<br>-When was first A-US?<br>-Was ultrasound machine portable or high frequency ultrasound<br>-Was fetal biometry used? If yes specify.<br>-What methods were used for GA cacluation<br>-Was quality and reliability was assessed? Intreobserver variabtion	-Who took LMP history?<br>-Are pregnant women aware of LMP?<br>-Were pregnant women has regular cycle?<br>-Not on contraception, 3 months before conception<br>-Breastfeed before conception<br>-Pregnancy complications<br>-When LMP was asked<br>-Method of assessing<br>-LMP Reliable

Table 3: Study characteristics

**Author & year**	**Year of publication**	**Continent**	**Reference standard for GA**	**Reference standard II**	**Reference standard III (PCS)**	**Eregie scoring model**	**Dubowitz Scoring**	**Ballard Scoring**	**Foot length for GA**	**Foot length for LBW**
Feresu SA, et al 2002 (27)	2002	Africa	Last menstrual period			-	√	√	-	-
Karunsekera KAW, et al 2002 (36)	2002	Asia	Antenatal ultrasound			-	√	-	-	-
Sunjoh F, et al 2004 (18)	2004	Africa	Last menstrual period			√	√	√	-	-
Mullany LC, et al, 2007 (61)	2007	Asia	-			-	-	-	-	√
Rosenberg RE, et al 2009 (38)	2009	Asia	Antenatal ultrasound			-	√	√	-	-
Marchant T, et al, 2010 (60)	2010	Africa	-		ESM	-	-	-	√	√
Taylor RAM, et al 2010 (42)	2010	Africa	Antenatal ultrasound			-	-	√	-	-
Alia RA, et al, 2011 (50)	2011	Asia	-			-	-	-	-	√
Rustagi N, et al, 2012 (56)	2012	Asia	-			-	-	-	-	√

Ahmed AA, et al, 2013	2013	Asia	-			-	-	-	-	√
Modibbo MH, et al, 2013 (53)	2013	Africa	-			-	-	-	-	√
Mukherjee S, et al, 2013 (24)	2013	Asia	-		BS	-	-	-	√	√
Nabiwemba E, et al 2013 (54)	2013	Africa	-		ESM	-	-	-	√	√
Thawani R, et al, 2013 (46)	2013	Asia	Last menstrual period			-	-	-	√	-
Wylie BJ, et al 2013 (5)	2013	Africa	Antenatal ultrasound			-	-	√	-	-
Otupiri E, et al, 2014 (55)	2014	Africa	-			-	-	-	-	√
Singhal S, et al, 2014 (45)	2014	Asia	Last menstrual period			-	-	-	√	-
Ashish KC, et al, 2015 (22)	2015	Asia	Last menstrual period			-	-	-	√	√
Moore KA, et al 2015 (37)	2015	Asia	Antenatal ultrasound			-	√	-	-	-
Srivastava A, et al, 2015 (58)	2015	Asia	-		BS	-	-	-	√	√
Thi HN, et al, 2015 (62)	2015	Asia	-		BS	-	-	-	√	√
Gavhane S, et al, 2016 (51)	2016	Asia	-		BS	-	-	-	√	√
Lee ACC, et al. 2016 (34)	2016	Asia	Antenatal ultrasound			√	-	√	√	-
Wyk V, et al, 2016 (48)	2016	Africa	Antenatal ultrasound		BS	-	-	-	√	-
Hadush MY, et al, 2017 (52)	2017	Africa	-			-	-	-	-	√
Pratinidhi AK, et al, 2017 (44)	2017	Asia	Last menstrual period			-	-	-	√	√
Singhal R, et al 2017 (41)	2017	Asia	Antenatal ultrasound			-	-	√	-	-
Srinivasa S, et al, 2017 (57)	2017	Asia	-		BS	-	-	-	√	√

Zahan GA, et al 2017 (28)	2017	Asia	Antenatal ultrasound	Last menstrual period		-	-	√	-	-
Rada S, et al 2018 (21)	2018	Africa	Last menstrual period			-	-	√	-	-
Paulsen CB, et al, 2019 (43)	2019	Africa	Antenatal ultrasound		BS	-	-	-	√	√
Unger H, et al 2019 (20)	2019	Both Asia & Africa	Antenatal ultrasound			-	-	√	-	-
Roy et al. 2019	2019	India	-	-	BS	-	-	-	√	-
Tenali et al. 2019	2019	India	-	-	BS	-	-	-	√	-
Gidi NW, et al, 2020 (59)	2020	Africa	-		ESM/BS	-	-	-	√	√
Trinche C, et al, 2020 (47)	2020	Africa	Last menstrual period			-	-	-	√	-
Kapoor A et al. 2020	2020	India	-	-	BS	-	-	-	√	-
Dagnew N et al. 2020	2020	Ethio pia	-	-	BS	-	-	-	√	-
Ajay K, et al. 2020	2020	India	-	-	BS	-	-	-	√	-
Rafat K, et al. 2020	2020	Egypt	-	-	BS	-	-	-	√	-
Srinavasa, et al. 2020	2020	India	-	-	BS	-	-	-	√	-
AMANHI Study Group, 2021 (39)	2021	Both Asia & Africa	Antenatal ultrasound			-	-	√	-	-
Raj M, et al 2021 (35)	2021	Asia	Antenatal ultrasound			√	-	√	-	-
Stevenson A, et al 2021 (23)	2021	Africa	Antenatal ultrasound			-	-	√	√	-
Tregstina M, et al. 2021	2021	Asia	Antenatal ultrasound			-	-	-	√	√
Pietravalle A, et al 2022	2022	Africa	Antenatal ultrasound			-	-	√	-	-
Ifa D. et al. 2023	2023	Africa	Last menstrual period			-	-	-	√	-

Mengi A, et al. 2023	2023	Ocean a	Antenatal ultrasound			-	-	-	√	√
Sintayehu E, et al. 2023	2023	Africa	Last menstrual period			-	-	-	√	√
Tikmani SS, et al. 2024	2024	Asia	Antenatal ultrasound			-	-	-	√	-

BS-Ballard scoring, DS-Dubowitz scoring, ESM-Eregie scoring model

Table 4: Characteristics of the studies that reported the validity of postnatal clinical examination versus antenatal ultrasound as the reference standard.

First author	Setting	Country	Reference standard	Clinical scoring	Study design	Sample size	Gestational age (Range in weeks)	Statistical test(s)
Rosenberg RE, et al. 2009	Hospital	Bangladesh	Ultrasound	BS & DS	Cohort study	355	Up to 33 weeks	-Mean difference<br>-Bland Altman plot 95% *CI<br>-Pearson correlation
Moore KA, et al. 2015	Hospital	Thai Myanmar border	Ultrasound	DS	Longitudinal cohort	250	16-40	-Mean difference<br>-Bland Altman plot 95% *CI<br>-Diagnostic accuracy
Lee ACC, et al. 2016	Community	Bangladesh	Ultrasound	BS & ESM	Study nested within-cluster randomized trial	1066	29.6–44.0	-Mean difference<br>-Bland Altman plot 95% *CI<br>-Diagnostic accuracy<br>-Pearson correlation
Raj M, et al. 2021	Hospital	India	Ultrasound	BS & ESM	Cross-sectional study	1114	26.3-41.0	-Mean difference

								-Bland Altman plot 95% *CI
Zahan GA, et al. 2017	Hospital	Bangladesh	Ultrasound	BS	Cross-sectional study	129	Not specified	-Correlation coefficient
Taylor RAM, et al. 2010	Community	Gambia	Ultrasound	BS	Cohort study	80	Not specified	-Correlation coefficient
Wylie BJ, et al. 2013	Community	Malawi	Ultrasound	BS	Cohort study	178	34 to 41	-Mean Difference SD, -Bland Altman 95% *CI
Stevenson A, et al. 2021	Hospital	South Africa	Ultrasound	BS	Prospective study	106	Not specified	-Mean Difference and SD -Correlation coefficient -Diagnostic accuracy -Bland Altman 95% *CI
Unger H, et al. 2019	Hospital	Multicountry study (Burkina Faso, Ghana, Malawi & Zambia)	Ultrasound	BS	Cohort study	1630	Not specified	-Mean Difference and SD -Correlation coefficient -Diagnostic accuracy -Bland Altman 95% *CI
AMANHI Study Group. 2021	Community	Multicountry study (Bangladesh, Ghana, Pakistan, Tanzania, and Zambia)	Ultrasound	BS	Cohort study	7428	34-<37	- Mean difference -Diagnostic accuracy -Bland Altman 95% *CI
Pietravalle A, et al 2022	Hospital	Tanzania	Ultrasound	BS	Retrospective	70	37-30	-Bland Altman
Karunsekera KAW, et al. 2002	Hospital	Sri Lanka	Ultrasound	DS	Cross-sectional study	200	35-42	-Mean difference

Table 5: characteristics of the studies that reported the validity of postnatal clinical examination versus last menstrual period (LMP) as reference standard.

First author	Setting	Country	Reference standard	Clinical scoring	Study design	Sample size	Gestational age (Range in weeks)	Statistical test(s)
Zahan GA, et al. 2017	Hospital	Bangladesh	LMP	BS	Cross-sectional study	129	Not specified	-Correlation coefficient
Feresu SA, et al. 2002	Hospital	Zimbabwe	LMP	BS, DS	Not available	364	28-40	-Pearson's correlation
Sunjoh F, et al. 2004	Hospital	Cameroon	LMP	BS, DS, ESM	Cross-sectional study	358	25-44	- Mean difference<br>- Pearson's correlation<br>- Bland Altman 95% *CI
Rada S, et al. 2018	Hospital	Multi-country study (Benin, Gabon, Mozambique, & Tanzania)	LMP	BS	Cohort study	4390	22-44	-Mean Difference and SD<br>-Bland Altman 95% *CI

**CI-Confidence interval*
***LMP-Last menstrual period*

Table 6: **Characteristics of the studies reported validity of foot length in assessing gestational age taking antenatal ultrasound or last menstrual period as reference standard**

Study	Setting	Country	Study design	Sample size	Landmarks	Instrument used	% Of preterm
**Antenatal Ultrasound as reference standard**							
Lee AC, et al. 2016	Community	Bangladesh	Cross-sectional study	710	heel to the tip of the big toe	Rigid plastic ruler	8.3
Wyk V, et al. 2016	Hospital	South Africa	Not specified	200	heel to the tip longest toe	Caliper	-
Paulsen CB, et al. 2019	Community	Tanzania	Observational study	376	heel to the tip longest toe	Rigid plastic ruler	-
Stevenson A, et al. 2021	Hospital	South Africa	Observational study	106	heel to the tip longest toe	Measuring tape	78.3
Tergestina M, et al. 2021	Hospital	India	Cross-sectional study	520	heel to the tip longest toe	Caliper	
Mengi A, et al. 2023	Clinics	Papua New Guinea	Prospective study	342	heal to tip of hallux	Rigid plastic ruler	7.3
Tikmani SS, et al. 2024	Community	Pakistan	Cross-sectional study	336	heal to tip of hallux	Rigid plastic ruler	22.4
**Last menstrual period as reference standard**							
Thawani R, et al. 2013	Hospital	India	Cross-sectional study	1000	heel to the tip longest toe	Caliper	37.3
Singhal S, et al. 2014	Hospital	India	Observational study	1000	heel to the tip of the big toe or second toe	Measuring tape	36.5
Ashish KC, et al. 2015	Hospital	Nepal	Cross-sectional study	811	heel to the tip of the big toe	Rigid plastic ruler	6.7
Pratinidhi AK, et al. 2017	Hospital	India	Not specified	645	heel to the tip longest toe	Caliper	-
Trinche C, et al. 2020	Hospital	Ethiopia	Cross-sectional study	424	heel to the tip of the big toe or second toe	Measuring tape	15.1
Ifa D, et al. 2023	Hospital	Ethiopia	Cross-sectional study	420	-	Measuring tape	16.4
Sintayehu E, et al. 2023	Hospital	Ethiopia	Cross-sectional study	381	heal to tip of hallux	Measuring tape	-
**Postnatal clinical scoring – Ballard scoring**							

Mukherjee S, et al. 2013	Hospital	India	Cross-sectional study	351	heel to the tip of the big toe	Rigid plastic ruler	48.1
Singhal S, et al. 2014	Hospital	India	Observational study	1000	heel to the tip of the big toe or second toe	Measuring tape (Steel)	15.4
Gavhane S, et al. 2016	Hospital	India	Observational study	800	heel to the tip longest toe	Caliper	15.5
Srivastava A, et al. 2015	Hospital	India	Not specified	254	heel to the tip of the big toe or second toe	Rigid plastic ruler	59.8
Thi HN, et al. 2015	Hospital	Vietnam	Observational study	485	heel to the tip of the big toe	Rigid plastic ruler	49%
Wyk V, et al. 2016	Hospital	South Africa	Not specified	200	heel to the tip longest toe	Caliper	-
Srinivasa S, et al. 2017	Hospital	India	Cross-sectional study	500	heel to the tip of the big toe or second toe	Rigid plastic ruler	16.8
Roy RA, et al. 2019	Hospital	India	Cross-sectional study	320	heel to the tip longest toe	Caliper	17.5
Tenali ASL, et al. 2019	Hospital	India	Prospective study	300	heel to the tip of the big toe	Caliper	28
Gidi NW, et al. 2020	Hospital	Ethiopia	Cross-sectional study	1389	heel to the tip of the big toe	Rigid plastic ruler	10.2
Kapoor A, et al. 2020	Hospital	India	Cross-sectional study	514	heel to the tip longest toe	Caliper	28.4
Dagnew N, et al. 2020	Hospital	Ethiopia	Cross-sectional study	-	heel to the tip of the big toe or second toe	Caliper	33.2
Ajay K, et al. 2020	Hospital	India	Cross-sectional study	350	heel to the tip of the big toe	Rigid plastic ruler	-
Rafat K, et al. 2020	Hospital	Egypt	Prospective study	1000	-	Caliper	-
Srinavasa S, et al. 2020	Hospital	India	Cross-sectional study	173	heel to the tip of the big toe or second toe	Rigid plastic ruler	29.5
**Postnatal clinical scoring (Eregie Scoring model)**							
Marchant T, et al. 2010	Hospital	Tanzania	Cross-sectional study	529	heel to the tip of the big toe	Rigid plastic ruler	9.0

Nabiwemba E, et al. 2013	Hospital	Uganda	Cross-sectional study	711	heel to the tip of the big toe	Rigid plastic ruler	4.0
Gidi NW, et al. 2020	Hospital	Ethiopia	Cross-sectional study	1389	heel to the tip of the big toe	Rigid plastic ruler	10.2

Table 7: Characteristics of studies reported validity of foot length in predicting low birth weight

First author	Setting	Country	Study design	Sample size	LBW, %	Statistical test
Hadush MY, et al. 2017	Hospital	Ethiopia	Cross-sectional study	422	27.0	-Pearson Correlation coefficients<br>-Diagnostic accuracy
Srinivasa S, et al. 2017	Hospital	India	Cross-sectional study	500	-	-Pearson Correlation coefficients<br>-Diagnostic accuracy
Thi HN, et al. 2015	Hospital	Vietnam	Prospective observational study	485	51.0	-Diagnostic accuracy
Otupiri E, et al. 2014	Hospital	Ghana	Cross-sectional study	973	21.7	-Pearson Correlation coefficients<br>-Diagnostic accuracy
Mullany LC, et al. 2007	Hospital	Nepal	Not specified	1640	28.6	-Diagnostic accuracy
Ashish KC, et al. 2015	Hospital	Nepal	Cross-sectional study	811	3.7	-Diagnostic accuracy
Nabiwemba E, et al. 2013	Hospital	Uganda	Cross-sectional study	711	12.0	-Diagnostic accuracy
Rustagi N, et al. 2012	Hospital	India	Prospective observational study	283	-	-Pearson Correlation coefficients<br>-Diagnostic accuracy
Gidi NW, et al. 2020	Hospital	Ethiopia	Cross-sectional study	1486	13.7	-Diagnostic accuracy
Paulsen CB, et al. 2019	Community	Tanzania	Prospective observational study	376	10.5	-Pearson Correlation coefficients<br>-Diagnostic accuracy
Ahmed AA, et al. 2014	Hospital	India	-	1028	-	-Correlation coefficients<br>-Diagnostic accuracy
Marchant T, et al. 2010	Hospital	Tanzania	Cross-sectional study	529	15.0	-Diagnostic accuracy
Mukherjee S, et al. 2013	Hospital	India	Cross-sectional study	351	51.8	-Pearson Correlation coefficients<br>-Diagnostic accuracy

Nabiwemba E, et al. 2013	Hospital	Uganda	Cross-sectional study	706	12.0	-Pearson Correlation coefficients<br>-Diagnostic accuracy
Alia RA, et al. 2011	Hospital	Bangladesh	Cross-sectional study	100	52.0	-Pearson Correlation coefficients
Gavhane S, et al. 2016	Hospital	India	Prospective observational study	800	25.5	-Correlation coefficients
Pratinidhi AK, et al. 2017	Hospital	India	-	645	-	-Correlation coefficients
Srivastava A, et al. 2015	Hospital	India	-	254	-	-Pearson Correlation coefficients
Modibbo MH, et al. 2013	Hospital	Nigeria	Cross-sectional study	551	-	-Pearson Correlation coefficients
Tregstina M, et al. 2021	Hospital	India	Cross-sectional study	520	-	-Correlation coefficients
Mengi A, et al. 2023	Hospital	Papua New Guinea	Prospective study	342	7.3	-Diagnostic accuracy
Sintayehu E, et al. 2023	Hospital	Ethiopia	Cross-sectional study	381	26.7	-Correlation coefficients

Figure 1: Quadas 2 summary (n-42)

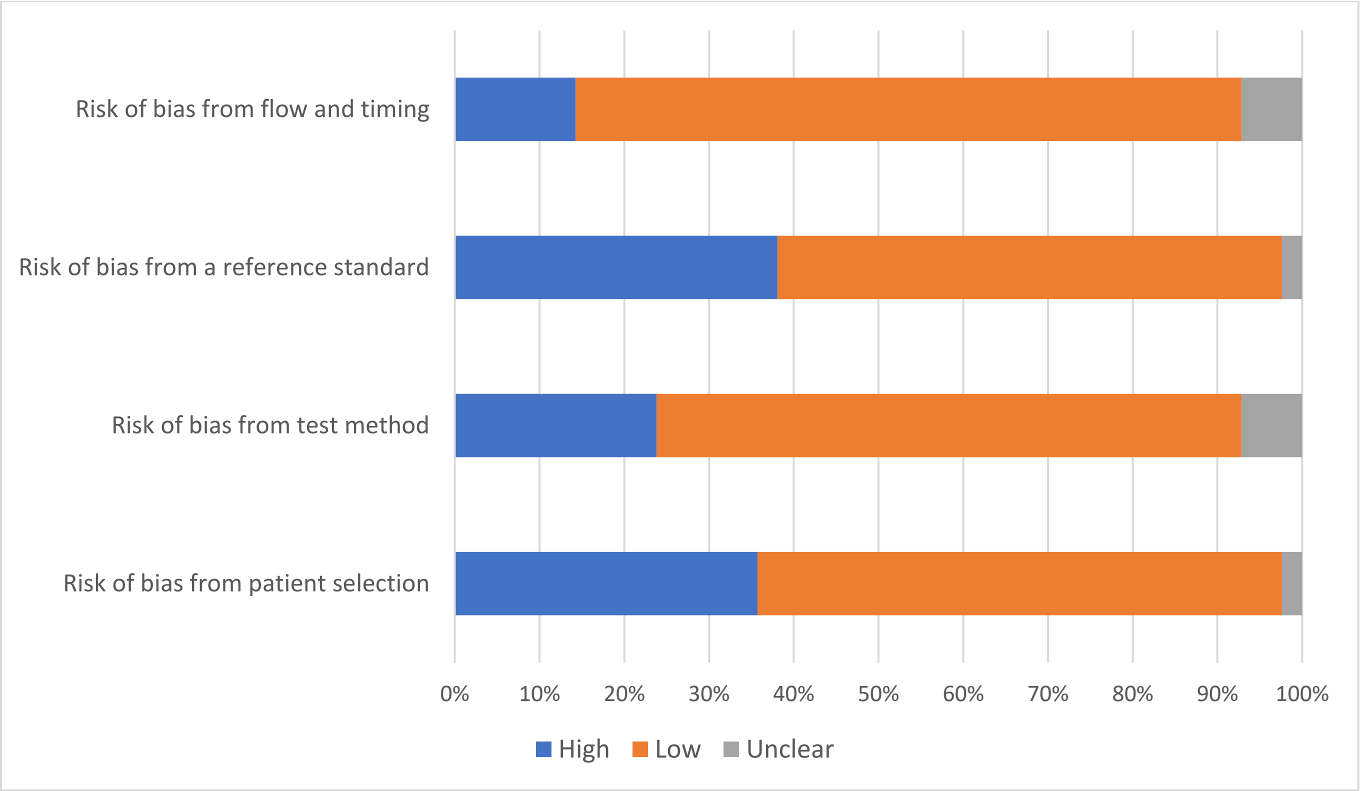

Figure 2: Figure 2: A random-effects forest plot comparing gestational age estimates by Ballard score versus the Last Menstrual Period, and antenatal ultrasound as the reference standard

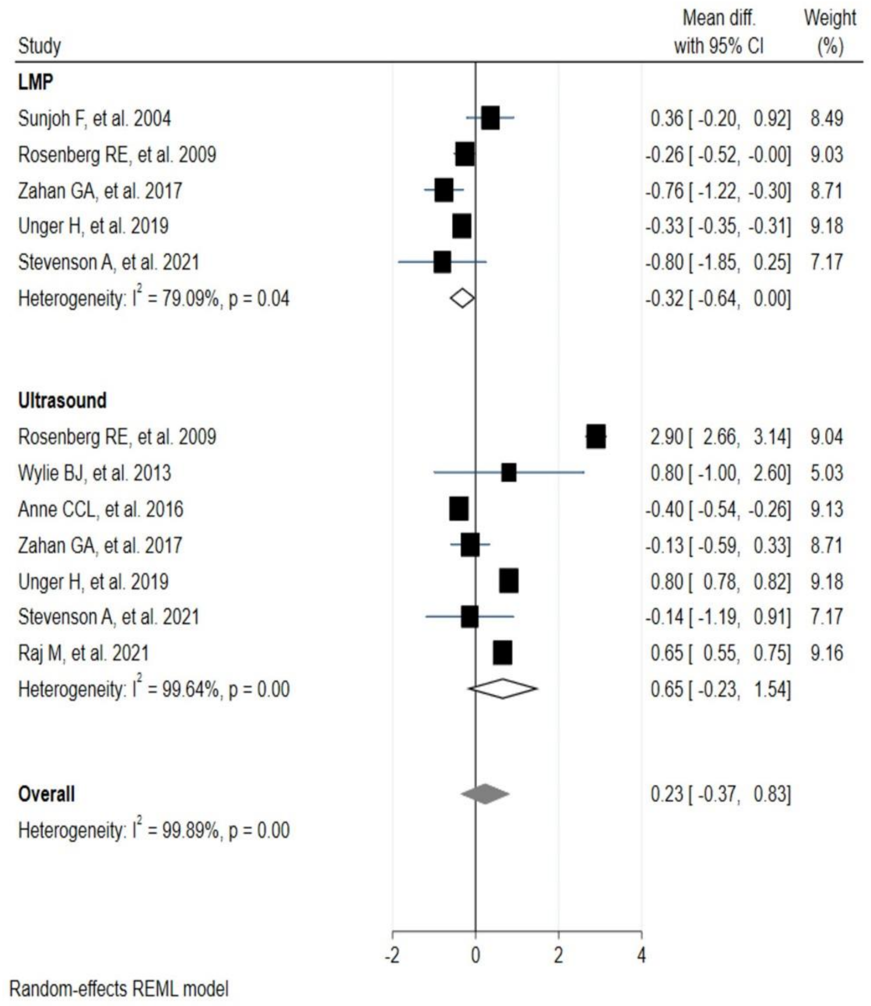

Figure 3: Pooled sensitivity and specificity of Ballard score for identification of preterm compared to antenatal ultrasound as the reference standard

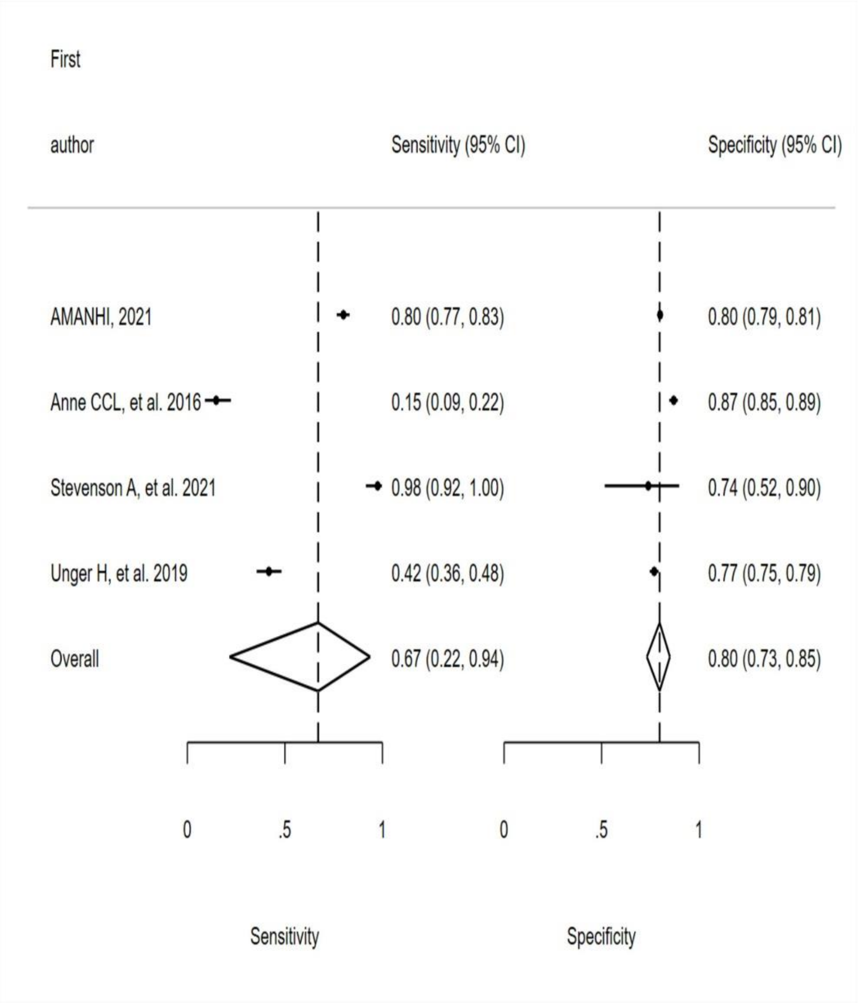

Figure 4: Pooled correlation coefficient between foot length and birth weight

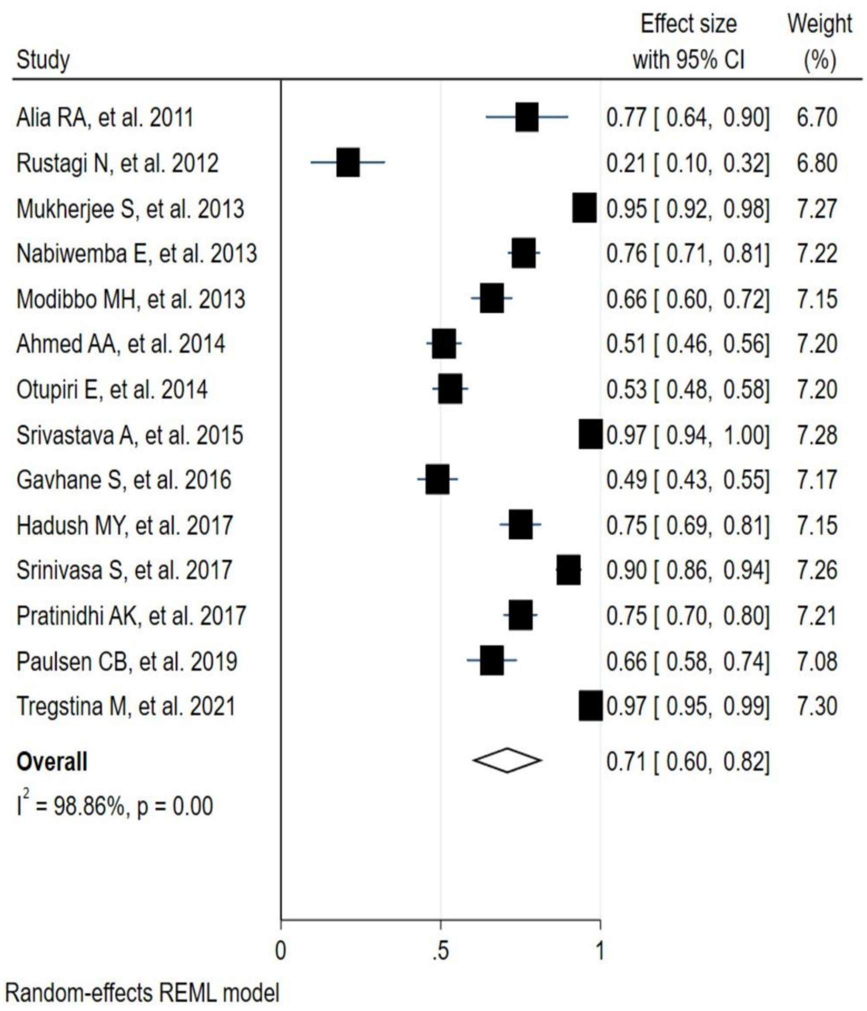

Supplement: online supplemental file 1 [file bmjpo-8-1-s001.pdf]
