# Supplementary material for: Assessing the diagnostic accuracy of postnatal clinical scoring methods and foot length measurement for estimating gestational age and birthweight of newborns in low- and middle-income countries: a systematic review and meta-analysis
Source: BMJ Paediatr Open. 2024 Aug 30;8(1):e002717. doi: 10.1136/bmjpo-2024-002717 (PMC11367336; doi:10.1136/bmjpo-2024-002717)
Supplement: Supplementary file 2 [file bmjpo-2024-002717.reviewer_comments.pdf]

PEER REVIEW HISTORY

BMJ Paediatrics Open publishes all reviews undertaken for accepted manuscripts. Reviewers are asked to complete a checklist review form and are provided with free text boxes to elaborate on their assessment. These free text comments are reproduced below.

ARTICLE DETAILS

**TITLE (PROVISIONAL)**	Assessing the Diagnostic Accuracy of Postnatal Clinical Scoring Methods and Foot Length measurements for estimating gestational age and birthweight of newborns in Low- and Middle-Income Countries: A systematic review and meta-analysis
**AUTHORS**	Tikmani, Shiyam Sunder<br>Mårtensson, Thomas<br>Khalid, Sumaira<br>Uzair, Muhammad<br>Ali, Qammerulanissa<br>Rahim, Anum<br>Mårtensson, andreas<br>Saleem, Sarah<br>brown, nick

VERSION 1 - REVIEW

**REVIEWER NAME**	Dr. nida zahid
**REVIEWER AFFILIATION**	Aga Khan University<br>Karachi<br>74800<br>Pakistan
**REVIEWER CONFLICT OF INTEREST**	No
**DATE REVIEW RETURNED**	13-May-2024

**GENERAL COMMENTS**	Introduction:<br>While the introduction outlines the limitations of antenatal ultrasound (A-US) in low- and middle-income countries (LMICs), it would be beneficial to elaborate further on why LMICs were chosen as the focus of the systematic review. This could include discussing the unique challenges faced by healthcare systems in LMICs regarding access to resources and the impact of these challenges on maternal and neonatal health outcomes. Moreover, the introduction provides a brief overview of postnatal clinical scoring (PCS) methods and foot length (FL) measurement as alternative approaches for estimating gestational age (GA) and birth weight. However, it could be enhanced by providing more details on the principles underlying these methods and their advantages and limitations compared to A-US and the last menstrual period (LMP). While the introduction outlines the purpose of the systematic review, a more explicit statement of the research objectives and the specific research questions addressed by the review would help to guide readers and provide clarity on the scope of the study.<br><br>Methods<br>The methods section of the systematic review is comprehensive and well-structured, providing detailed information on the search strategy, inclusion criteria, data extraction procedure, quality assessment, and data analysis

	methods. However, a few areas could be further refined for clarity and completeness:<br>Inclusion and Exclusion Criteria: The inclusion and exclusion criteria are clearly defined, but it would be helpful to provide rationale or justification for each criterion to clarify why certain studies were included or excluded. For instance, the decision to exclude studies assessing gestational age on or after day 7 post-birth could be explained in more detail.<br>Data Extraction Procedure: The data extraction procedure is described in detail, including the information extracted from each study. However, it would be useful to specify how discrepancies between reviewers were resolved during the screening and extraction process to ensure consistency and minimize bias.<br>Quality Assessment: The use of the QUADAS-2 tool for assessing the methodological quality of included studies is appropriate. It is commendable that disagreements between reviewers were resolved through discussion or consultation with a third reviewer. However, it would be beneficial to provide more information on how the QUADAS-2 tool was applied, including any specific criteria used to assign scores for each domain.<br><br>Results : The results section provides a detailed overview of the included studies and their findings regarding the diagnostic accuracy of postnatal clinical scoring (PCS) methods and foot length (FL) for estimating gestational age (GA) and identifying preterm birth and low birth weight (LBW).<br><br>Discussion: the implications of these findings for clinical practice and future research could be further elaborated. For example, discussing the potential impact of overestimating or underestimating GA on clinical decision-making and neonatal outcomes would enhance the practical relevance of the findings.

**REVIEWER NAME**	Dr. Peter Rohloff
**REVIEWER AFFILIATION**	2 Calle 5-43 Zona 1<br>Santiago Sacatepéquez<br>3006<br>Guatemala
**REVIEWER CONFLICT OF INTEREST**	No
**DATE REVIEW RETURNED**	13-May-2024

**GENERAL COMMENTS**	This is an important topic but I have a number of concerns that limit my enthusiasm for this paper.<br><br>First, there have been a number of systematic reviews on this topic, including several very recent ones, For example the BMJ Global Health review by Lee and colleagues from 2020 includes most of the same data. These reviews are not interacted with critically and it is unclear why another review is needed at this time.<br><br>https://gh.bmj.com/content/5/11/e002976<br>https://pubmed.ncbi.nlm.nih.gov/36774458/<br>https://pubmed.ncbi.nlm.nih.gov/29150458/<br><br>Second, the review has a clear interest in demonstrating that FL is an important emerging strategy, which it likely is, but these leads to a bias in presentation of results that overemphasize the importance of FL. Given the

	extreme heterogeneity in the studies reviewed and the methods utilized this systematic review doesn't help advance the conclusions stated that "Foot length emerges as a potential tool for augmenting the identification of high-risk neonates" any more than any of the original included studies.<br><br>A major limitation of all of the work on this topic is that the methods used are not standardized, and this limits the ability to draw meta inference. The BMJ GH review does not perform meta-analysis, for example, because of this. The I2 statistics here are >95%, so I question whether the meta-analysis presented is useful at all, even with a random effects model. More so because it is only for the Ballard.<br><br>Conclusions like "CS methods such as BS and DS tend to overestimate GA" cannot be justified from the data. If we look at the authors' Figure 2 for the Ballard we see striking heterogeneity and we also see that the estimates are in opposite directions for LMP based studies vs ultrasound based studies. So all we can actually conclude here is that there is a lot of variability or measurement error in the reference standard used (LMP or Ultrasound) and no clear conclusions can be drawn. Until methods are better standardized in this field across multiple countries and initiatives I fear that drawing more meaningful conclusions will remain elusive.<br><br>Another major limitation of the usefulness of this synthesis is that the clinical scoring systems are not directly compared to foot length. This is, in fact, the question that really needs to be answered, how do different post natal methods compare to each other, rather than parallel comparison of different post natal methods to antenatal measures.<br><br>Given the fact that most of the observed variation and equivocal findings in the synthesis are due to major differences in methodology, it would be useful to see this compared in more detail. In fact, I think this would be the unique contribution of another systematic review, but this has not been done here. So, for example, what matters is not that a study did antenatal ultrasound as the reference standard, but rather , the details of how and when that ultrasound was performed (type of training or technician matters, as does age at ultrasound - 14 wks? 20 wks? Late in pregnancy?; portable vs higher grade ultrasound? BMI of enrolled subjects which greatly influences diagnostic accuracy of antenatal ultrasound especially with portable devices?). Similarly, not just if LMP was used but how it was ascertained and verified. Was LMP considered reliable or not (factors which would make LMP not reliable would include all of the following: not having regular menstruation in months prior to pregnancy; not using hormonal therapy in months prior to pregnancy; not breastfeeding immediately prior to conception, and not recently pregnant). Finally, the authors note significant differences in how FL is assessed among studies, but what would be most interesting here is comparison of how FL functions diagnostically as a function of these methodological differences.<br><br>An additional methodological issue with almost all the FL studies that limits conclusions is that outpoints are always used (LBW vs not LBW; preterm vs not preterm). At the formative stage of trying to figure out if different test methods are systematically biased (Bland Altman plots) what is really needed is an analysis of these tests across the continuous variable (GA or weight), as test performance may not be the same across the entire range of the variable of interest. I feel that what is really needed is a pooled reanalysis of all individual level data from these studies, rather than a summary meta analysis that can't get to these questions.

	In summary, I would say that the state of the literature in this field is greatly hampered by methodological differences between studies which completely obstruct confident conclusions. This systematic review presents summative statements which are of low confidence because of those differences, and it also does not clearly present these differences in a way that could help change the way such studies are being done.

**REVIEWER NAME**	Dr. Shanti Raman
**REVIEWER AFFILIATION**	South Western Sydney Local Health District<br>Department of Community Paediatrics<br>Health Services Building Level 3, Cnr Campbell & Goulburn St,<br>Liverpool<br>New South Wales<br>2170<br>Australia
**REVIEWER CONFLICT OF INTEREST**	None
**DATE REVIEW RETURNED**	24-May-2024

**GENERAL COMMENTS**	General: This is an important topic and I commend the authors for exploring this issue in detail. There is however a feeling- of “so what”; much of this information is already known, even if foot length measurements are carried out accurately to detect LBW and/prematurity, there is still a need to continue to track these infants’ growth effectively and accurately in the community. Additionally, it is not clear, how much added value there is from this systematic review, given the other recent systematic reviews done on this topic in the recent past. The title should be Assessing the Diagnostic Accuracy of Postnatal Clinical Scoring Methods and Foot Length measurements for estimating gestational age and birthweight of newborns in Low- and Middle-Income Countries: A systematic review and meta-analysis<br>Abstract: Don’t repeat “This systematic review” in the Background and Methods. Perhaps replace the Background statement with: We therefore wanted to assess the diagnostic accuracy of the postnatal clinical scoring (PCS) methods/models of Ballard (BS), Dubowitz (DS), and Eregie (ESM) and foot length (FL) for estimating gestational age (GA) and birth weight to identify preterm birth and LBW compared to antenatal ultrasound (A-US) and digital weighing scales as reference standard in LMICs. Methods: say “We searched” rather than This systematic review searched.<br>Material and Methods: Authors state: building upon a previous review that examined studies up to June 2022- has this review been published, if so needs to be referenced.<br>Discussion<br>While authors make the point that FL appears to show promise as a tool for augmenting the identification of high-risk neonates in Low- and Middle-Income Countries, they do not comment on previous review findings that state FL can identify babies of low birthweight (but not preterm birth) with high sensitivity, though somewhat lower specificity. Also, about the differences between measurement cut-offs in African versus Asian studies.<br>In Conclusion- is there enough evidence from this systematic review- to suggest FL measurements in hospital and community settings can help identify LBW and preterm babies accurately? If not, this has to be stated. Also, about what this means for clinical p

VERSION 1 – AUTHOR RESPONSE

On behalf of all authors, I express my sincere gratitude for your thorough and insightful review of our research paper. Your valuable feedback has significantly enhanced the quality of our work, and we truly appreciate the time and expertise you dedicated to the process. Your contributions are invaluable, and we are grateful for your commitment to advancing academic excellence.
We've made revisions using the "Track Changes" function in the MS Word document, clearly marked as such. Additionally, we've included a clean copy following the acceptance of track changes.
Reviewer: 2
Comments to the Author
This is an important topic but I have a number of concerns that limit my enthusiasm for this paper.

First, there have been a number of systematic reviews on this topic, including several very recent ones, For example the BMJ Global Health review by Lee and colleagues from 2020 includes most of the same data. These reviews are not interacted with critically and it is unclear why another review is needed at this time.

https://gh.bmj.com/content/5/11/e002976
https://pubmed.ncbi.nlm.nih.gov/36774458/
https://pubmed.ncbi.nlm.nih.gov/29150458/

Author's response:
We acknowledge previous systematic reviews on this topic, including those by Lee et al. in 2017 (clinical signs) and Folger et al. in 2020 (foot length). These reviews included studies up to June 2016 and May 2020, respectively, and identified issues with study quality and high heterogeneity, recommending further research using antenatal ultrasound as a reference standard. Since then, six studies on postnatal clinical scoring and five studies on foot length have been published. Incorporating these recent studies allows us to update and refine gestational age estimates. Additionally, while previous reviews discussed the quality of reference standards, our review quantifies this aspect, enabling a more robust meta-analysis. These points have been included in the introduction of the revised manuscript.
Second comment
The review has a clear interest in demonstrating that FL is an important emerging strategy, which it likely is, but these leads to a bias in presentation of results that overemphasize the importance of FL. Given the extreme heterogeneity in the studies reviewed and the methods utilized this systematic review doesn't help advance the conclusions stated that "Foot length emerges as a potential tool for augmenting the identification of high-risk neonates" any more than any of the original included studies.
Author's response:
Thank you for your thoughtful feedback. We understand your concern regarding the potential bias in the presentation of results, particularly in overemphasizing the importance of foot length (FL). We carefully addressed this issue in the revised version of the manuscript by ensuring a balanced presentation of the findings and their implications. Additionally, we provided more explicit clarity on the limitations associated with the extreme heterogeneity observed in the reviewed studies and their impact on the conclusions drawn.

Third comment
A major limitation of all of the work on this topic is that the methods used are not standardized, and this limits the ability to draw meta inference. The BMJ GH review does not perform meta-analysis, for example, because of this. The I2 statistics here are >95%, so I question whether the meta-analysis presented is useful at all, even with a random effects model. More so because it is only for the Ballard.
Author's comments:
Thank you for your observation. The lack of standardized methods and high heterogeneity ($I^2 > 95\%$) present significant challenges in our review. However, our meta-analysis remains valuable as it

quantifies this heterogeneity, emphasizing the need for standardized approaches. Despite the challenges, our analysis provides valuable insights into the variability of gestational age estimation. We focused on the Ballard score due to the availability of sufficient data, while the Dubowitz and Eregie models lacked enough data for a robust meta-analysis. Addressing these methodological inconsistencies is vital for enhancing the reliability and accuracy of future gestational age assessments.

Fourth comment
Conclusions like “CS methods such as BS and DS tend to overestimate GA” cannot be justified from the data. If we look at the authors’ Figure 2 for the Ballard we see striking heterogeneity and we also see that the estimates are in opposite directions for LMP based studies vs ultrasound based studies. So all we can actually conclude here is that there is a lot of variability or measurement error in the reference standard used (LMP or Ultrasound) and no clear conclusions can be drawn. Until methods are better standardized in this field across multiple countries and initiatives I fear that drawing more meaningful conclusions will remain elusive.
Author’s response:
Your insightful observation is duly noted. Upon careful examination of the data, it's apparent that conclusions suggesting clinical scoring (CS) methods like the Ballard Score (BS) and Dubowitz Score (DS) tend to overestimate gestational age (GA) may not be entirely supported. Consequently, drawing definitive conclusions from the current data is challenging. We agree that drawing more meaningful conclusions may remain elusive until methods are standardized across various countries and initiatives. To address this concern, we have added a separate objective to assess the quality of the reference standards. Based on specific criteria for both the reference and index tests, we will redo the meta-analysis to assess the heterogeneity more comprehensively. We have ensured a balanced approach in interpreting the results and have addressed these issues in the revised manuscript. We have incorporated suggested changes in the revised manuscript.

Fifth comment:
Another major limitation of the usefulness of this synthesis is that the clinical scoring systems are not directly compared to foot length. This is, in fact, the question that really needs to be answered, how do different post natal methods compare to each other, rather than parallel comparison of different post natal methods to antenatal measures.
Author’s response:
Your observation is indeed important and warrants attention. Comparing the gold standards, which are antenatal ultrasound, and the last menstrual period (LMP) with clinical scoring or foot length is crucial for gestational age (GA) assessment. This comparison provides valuable insights into the accuracy and reliability of postnatal clinical scoring systems. Additionally, we acknowledge the limitation regarding comparing clinical scoring systems with foot length, as direct comparisons between various postnatal methods are essential for determining their relative efficacy and reliability. The primary objective of the review was to compare clinical scoring and foot length with the gold standard. To ensure consistency, we have adhered to the stated objective of the study.
Sixth comment:
Given the fact that most of the observed variation and equivocal findings in the synthesis are due to major differences in methodology, it would be useful to see this compared in more detail. In fact, I think this would be the unique contribution of another systematic review, but this has not been done here. So, for example, what matters is not that a study did antenatal ultrasound as the reference standard, but rather , the details of how and when that ultrasound was performed (type of training or technician matters, as does age at ultrasound - 14 wks? 20 wks? Late in pregnancy?; portable vs higher grade ultrasound? BMI of enrolled subjects which greatly influences diagnostic accuracy of antenatal ultrasound especially with portable devices?). Similarly, not just if LMP was used but how it was ascertained and verified. Was LMP considered reliable or not (factors which would make LMP not reliable would include all of the following: not having regular menstruation in months prior to pregnancy; not using hormonal therapy in months prior to pregnancy; not breastfeeding immediately prior to conception, and not recently pregnant). Finally, the authors note significant differences in how FL is assessed among studies, but what would be most interesting here is comparison of how FL functions diagnostically as a function of these methodological differences.

Author's response:
Thank you for your suggestion. Due to the variations in methodology across studies, comparing these details in more depth could indeed offer a unique contribution to another systematic review. In the revised version, we provided a more detailed comparison of the methodology, including how and when antenatal ultrasounds and LMP were performed and verified, as well as how FL functions diagnostically as a function of these methodological differences. We have incorporated a table comparing the reference standards (Antenatal ultrasound and LMP) based on parameters suggested by the reviewer. The changes were made in the revised manuscript.

Seventh comment:
An additional methodological issue with almost all the FL studies that limits conclusions is that outpoints are always used (LBW vs not LBW; preterm vs not preterm). At the formative stage of trying to figure out if different test methods are systematically biased (Bland Altman plots) what is really needed is an analysis of these tests across the continuous variable (GA or weight), as test performance may not be the same across the entire range of the variable of interest. I feel that what is really needed is a pooled reanalysis of all individual level data from these studies, rather than a summary meta-analysis that can't get to these questions.
Author's response:
Your observation is indeed crucial, especially in LMICs and rural settings where dichotomized outcomes are significant for practical clinical decision-making. This focus is justified given the pressing needs of these contexts. Additionally, the use of dichotomized outcomes in many foot length (FL) studies limits conclusions due to different cut-points, as well as when assessing systematic biases across the entire range of the variable of interest. However, pooling individual-level data from these studies is not feasible due to various methodological and practical constraints. We will mention this limitation in the discussion section of the revised version of our systematic review. We have addressed those comments in the limitations of the review.

Eighth comment
In summary, I would say that the state of the literature in this field is greatly hampered by methodological differences between studies which completely obstruct confident conclusions. This systematic review presents summative statements which are of low confidence because of those differences, and it also does not clearly present these differences in a way that could help change the way such studies are being done.
Author's response:
We greatly appreciate the time and thought you have given to your feedback and will address comments in the various sections of the revised manuscript to enhance the clarity and confidence in the conclusions drawn in our review.

Reviewer: 3
Comments to the Author
General: This is an important topic and I commend the authors for exploring this issue in detail. There is however a feeling- of "so what"; much of this information is already known, even if foot length measurements are carried out accurately to detect LBW and/prematurity, there is still a need to continue to track these infants' growth effectively and accurately in the community. Additionally, it is not clear, how much added value there is from this systematic review, given the other recent systematic reviews done on this topic in the recent past. The title should be Assessing the Diagnostic Accuracy of Postnatal Clinical Scoring Methods and Foot Length measurements for estimating

gestational age and birthweight of newborns in Low- and Middle-Income Countries: A systematic review and meta-analysis
Author's response:
Thank you for your valuable feedback and for acknowledging the importance of the topic. We appreciate your commendation and would like to address your concerns. We agree that even with accurate foot length measurements, continuous tracking of infant growth in the community remains crucial. However, our systematic review aims to provide a comprehensive assessment of the diagnostic accuracy of postnatal clinical scoring methods and foot length measurements specifically for estimating gestational age and birth weight in low- and middle-income countries (LMICs). We believe that our review adds value by integrating and updating existing findings with more recent data and addressing methodological limitations and biases. Regarding your suggestion for the title, we will modify it as follows: "Assessing the Diagnostic Accuracy of Postnatal Clinical Scoring Methods and Foot Length Measurements for Estimating Gestational Age and Birthweight of Newborns in Low- and Middle-Income Countries: A Systematic Review and Meta-Analysis." We have significantly revised introduction, results, discussion/implications/limitations and conclusions.

Abstract: Don't repeat "This systematic review" in the Background and Methods. Perhaps replace the Background statement with: We therefore wanted to assess the diagnostic accuracy of the postnatal clinical scoring (PCS) methods/models of Ballard (BS), Dubowitz (DS), and Eregie (ESM) and foot length (FL) for estimating gestational age (GA) and birth weight to identify preterm birth and LBW compared to antenatal ultrasound (A-US) and digital weighing scales as reference standard in LMICs. Methods: say "We searched" rather than This systematic review searched.
Material and Methods: Authors state: building upon a previous review that examined studies up to June 2022- has this review been published, if so needs to be referenced.
Author's response:
We revised the manuscript based on your kind feedback.
Discussion
While authors make the point that FL appears to show promise as a tool for augmenting the identification of high-risk neonates in Low- and Middle-Income Countries, they do not comment on previous review findings that state FL can identify babies of low birthweight (but not preterm birth) with high sensitivity, though somewhat lower specificity. Also, about the differences between measurement cut-offs in African versus Asian studies. In Conclusion- is there enough evidence from this systematic review- to suggest FL measurements in hospital and community settings can help identify LBW and preterm babies accurately? If not, this has to be stated. Also, about what this means for clinical practice out in the community for further tracking the growth and wellbeing of infants at risk.
Author's feedback
Thank you for your insightful comment. We acknowledge the importance of discussing previous review findings regarding foot length (FL) measurements and we have added them in introduction as well as in the discussion. Regarding your question about whether there is enough evidence from this systematic review to suggest FL measurements in hospital and community settings can accurately identify LBW and preterm babies, we emphasized the need for further research to determine the accuracy and practical implications of FL measurements in different settings. Additionally, we discussed the implications of our findings for clinical practice in the community, particularly in terms of tracking the growth and well-being of infants at risk.

VERSION 2 - REVIEW

**REVIEWER NAME**	Dr. Peter Rohloff
**REVIEWER AFFILIATION**	2 Calle 5-43 Zona 1<br>Santiago Sacatepéquez<br>3006<br>Guatemala
**REVIEWER CONFLICT OF INTEREST**	No
**DATE REVIEW RETURNED**	13-May-2024

**GENERAL COMMENTS**	The paper is much improved, with the addition of the quality assessments

	and moderation of the conclusions. I would recommend making sure the metanalysis is checked by one of BMJPO stats reviewers with expertise in metanalysis, but otherwise feel that the paper is in much better shape.

**REVIEWER NAME**	Dr. Shanti Raman
**REVIEWER AFFILIATION**	South Western Sydney Local Health District<br>Department of Community Paediatrics<br>Health Services Building Level 3, Cnr Campbell & Goulburn St,<br>Liverpool<br>New South Wales<br>2170<br>Australia
**REVIEWER CONFLICT OF INTEREST**	None
**DATE REVIEW RETURNED**	24-May-2024
**GENERAL COMMENTS**	General: Thanks for making the critical changes to your paper, which has strengthened it and not overstated the significance of one measure which is foot length. I think this is much improved. There is still some convincing needed however, that this review adds to new knowledge and therefore some policy and practice implications. There needs to be careful review of language, grammar and style- some idiosyncratic use of Capitalising (UPPERCASE) for example.<br>What do the new findings imply:<br>Typo here in 1st statement, so rephrase to: There is an urgent need for standardized GA and birth weight measurement protocols, as well as consensus on reference standards, to improve the reliability and accuracy of PCS and FL assessments in LMICs.<br>Introduction<br>State: The objectives of this study were therefore: 1) to update the existing systematic reviews and meta-analyses on diagnostic accuracy of PCS and FL for GA and birth weight assessment in a single review in the LMIC context, and 2) to assess the quality of evidence related to reference standards of: i) Antenatal ultrasound and ii) Last menstrual period.<br>Results<br>This section needs a careful editing for language. A few missing punctuation marks and many grammatical errors.<br>Quality assessment P8: Studies used LMP as a reference standard showed high risk of bias, change to “Studies using LMP as a reference standard showed high risk of bias”<br>Also, when reporting on one study’s test characteristics- P9, just report “with sensitivity of --- and specificity of ---, instead of: “has sensitivity was 32.7%, and specificity was”.<br>QUALITY OF THE REFERENCE STANDARDS FOR GESTATIONAL AGE AND BIRTH WEIGHT. Does not have to be in UPPERCASE, remove “the”<br>DIAGNOSTIC ACCURACY OF FOOT LENGTH IN ASSESSING THE LOW BIRTH WEIGHT,<br>Why is this title in uppercase? Should be: Diagnostic Accuracy of Foot Length in Assessing Low Birth Weight (no comma)

	DS resulted in a pooled mean difference was 0.68- should this not be "DS resulted in a pooled mean difference of 0.68"?<br>Discussion<br>The authors could comment on the differences between South Asian studies- where the bulk of premature and low birth weight babies occur, and other settings, especially Africa- where perhaps different cut-offs may even apply. This adds to the contextual complexity of using supposedly 'simple' tools to evaluate low birth weight and prematurity.<br>Conclusion<br>In the context of low resource settings, ongoing growth monitoring in the early years is actually a major concern. So even if progress is made in addressing accurate estimation of prematurity and low birth weight, there is a need to upskill

**REVIEWER NAME**	Dr. Javier Ballesteros
**REVIEWER AFFILIATION**	University of the Basque Country<br>Dept of Neuroscience - Psychiatry<br>Barrio Sarriena S/N<br>Medical School<br>Leioa<br>Vizcaya<br>48690<br>Spain
**REVIEWER CONFLICT OF INTEREST**	No
**DATE REVIEW RETURNED**	16-Jul-2024
**GENERAL COMMENTS**	Review of manuscript bmjpo-2024-002717.R1<br><br>This manuscript presents a systematic review and meta-analysis previously registered in PROSPERO with the code CRD42020209455. My comments refer only to the issues related to the methods used for systematic review and meta-analysis, and their interpretation. I do not see any problem with the methods followed in the manuscript. They fit well with the study's aims and the methods underlined on the PROSPERO registration. The only point that merits a point in the manuscript's discussion is the discrepancies between the PROSPERO's protocol and this manuscript, if any.

VERSION 2 – AUTHOR RESPONSE

Reviewer: 2
Dr. Peter Rohloff
Comments to the Author
The paper is much improved, with the addition of the quality assessments and moderation of the conclusions. I would recommend making sure the metanalysis is checked by one of BMJPO stats reviewers with expertise in metanalysis, but otherwise feel that the paper is in much better shape.
Response:
Thank you for your critical review and valuable feedback.

Reviewer: 3
Dr. Shanti Raman, South Western Sydney Local Health District, University of New South Wales
Comments to the Author

General: Thanks for making the critical changes to your paper, which has strengthened it and not overstated the significance of one measure which is foot length. I think this is much improved.
Response:
Thank you for the guidance and providing the opportunity to revise this manuscript.

There is still some convincing needed however, that this review adds to new knowledge and therefore some policy and practice implications.
Response
Thank you. We have added the statement regarding policy and practice implication.

There needs to be careful review of language, grammar and style- some idiosyncratic use of Capitalising (UPPERCASE) for example. We have carefully reviewed the text and addressed punctuation, grammar and spelling errors.
What do the new findings imply:
Typo here in 1st statement, so rephrase to: There is an urgent need for standardized GA and birth weight measurement protocols, as well as consensus on reference standards, to improve the reliability and accuracy of PCS and FL assessments in LMICs.
Response
Thank you, the statement was rephrased.

Introduction
State: The objectives of this study were therefore: 1) to update the existing systematic reviews and meta-analyses on diagnostic accuracy of PCS and FL for GA and birth weight assessment in a single review in the LMIC context, and 2) to assess the quality of evidence related to reference standards of: i) Antenatal ultrasound and ii) Last menstrual period.
Response
Thank you, the objectives were revised.
Results
This section needs careful editing for language. A few missing punctuation marks and many grammatical errors.
Response:
We carefully reviewed the language and adjusted grammar and punctuation errors.

Quality assessment P8: Studies used LMP as a reference standard showed high risk of bias, change to "Studies using LMP as a reference standard showed high risk of bias"
Response:
Thank you so much. Statement changed accordingly.

Also, when reporting on one study's test characteristics- P9, just report "with sensitivity of --- and specificity of ---, instead of: "has sensitivity was 32.7%, and specificity was". Thank you. As advised, we carefully reviewed this and make necessary amendments.
QUALITY OF THE REFERENCE STANDARDS FOR GESTATIONAL AGE AND BIRTH WEIGHT. Does not have to be in UPPERCASE, remove "the" Thank you. We used appropriate case style.
DIAGNOSTIC ACCURACY OF FOOT LENGTH IN ASSESSING THE LOW BIRTH WEIGHT,
Response
Thank you. We used appropriate case style.

Why is this title in uppercase? Should be: Diagnostic Accuracy of Foot Length in Assessing Low Birth Weight (no comma)
Response:
Thank you. We used an appropriate case style.

DS resulted in a pooled mean difference was 0.68- should this not be "DS resulted in a pooled mean difference of 0.68"?
Response:
Thank you so much. The statement changed accordingly.

Discussion
The authors could comment on the differences between South Asian studies- where the bulk of premature and low birth weight babies occur, and other settings, especially Africa- where perhaps different cut-offs may even apply. This adds to the contextual complexity of using supposedly 'simple' tools to evaluate low birth weight and prematurity.
Response:
Thank you. We have added the response in discussion.

Conclusion
In the context of low resource settings, ongoing growth monitoring in the early years is actually a major concern. So even if progress is made in addressing accurate estimation of prematurity and low birth weight, there is a need to upskill health workers to accurately measure and monitor growth of infants and toddlers in the community.

Response:
Thank you so much. Statement changed accordingly.

Reviewer: 4
Dr. Javier Ballesteros, University of the Basque Country
Comments to the Author
Review of manuscript bmjpo-2024-002717.R1

This manuscript presents a systematic review and meta-analysis previously registered in PROSPERO with the code CRD42020209455. My comments refer only to the issues related to the methods used for systematic review and meta-analysis, and their interpretation. I do not see any problem with the methods followed in the manuscript. They fit well with the study's aims and the methods underlined on the PROSPERO registration. The only point that merits a point in the manuscript's discussion is the discrepancies between the PROSPERO's protocol and this manuscript, if any.
Response
Thank you. There is a deviation from the protocol. We have added this statement below the limitations
